# Ribosome-induced RNA conformational changes in a viral 3′-UTR sense and regulate translation levels

**DOI:** 10.1038/s41467-018-07542-x

**Published:** 2018-11-29

**Authors:** Erik W. Hartwick, David A. Costantino, Andrea MacFadden, Jay C. Nix, Siqi Tian, Rhiju Das, Jeffrey S. Kieft

**Affiliations:** 1 Department of Biochemistry and Molecular Genetics, University of Colorado Denver School of Medicine, Aurora, CO 80045 USA; 2 RNA BioScience Initiative, University of Colorado Denver School of Medicine, Aurora, CO 80045 USA; 3 Molecular Biology Consortium, Advanced Light Source, Lawrence Berkeley National Laboratory, Berkeley, CA 94720 USA; 4 Department of Biochemistry, Stanford University, Stanford, CA 94305 USA

## Abstract

Structured RNA elements, programmed RNA conformational changes, and interactions between different RNA domains underlie many modes of regulating gene expression, mandating studies to understand the foundational principles that govern these phenomena. Exploring the structured 3′ untranslated region (UTR) of a viral RNA, we discovered that different contexts of the 3′-UTR confer different abilities to enhance translation of an associated open reading frame. In one context, ribosome-induced conformational changes in a ‘sensor’ RNA domain affect a separate RNA ‘functional’ domain, altering translation efficiency. The structure of the entire 3′-UTR reveals that structurally distinct domains use a spine of continuously stacked bases and a strut-like linker to create a conduit for communication within the higher-order architecture. Thus, this 3′-UTR RNA illustrates how RNA can use programmed conformational changes to sense the translation status of an upstream open reading frame, then create a tuned functional response by communicating that information to other RNA elements.

## Abstract (web summary)

Complex RNA three-dimensional structures undergo functionally important programmed conformational changes. Here, the authors report how two structurally and functionally coupled RNA domains within a viral 3′-UTR sense the ribosome through conformational changes and respond by modulating translation.

## Introduction

Eukaryotic regulation of gene expression at the level of translation can be controlled by structured RNA elements within messenger RNAs (mRNAs) that alter translation initiation^1,2^, adjust protein coding potential^3,4^, and affect RNA chemical stability^5–7^. These elements can be found throughout an mRNA or viral RNA, in both the 5′- and 3′-untranslated regions (UTRs) as well as in the protein coding sequences. In addition, RNA conformational changes, structural cooperation between different RNA domains, or the context of the RNA element potentially could combine to enhance regulatory potential and capacity. However, many underlying principles that allow complex higher-order RNA structure-based regulation remain poorly understood as few examples have been characterized in detail. Viruses, particularly single-stranded positive-sense RNA viruses, contain a variety of structured RNA regulatory elements that interact with and exploit the cellular machinery and thus they provide a wealth of RNAs that serve as models to yield insight into basic principles of diverse and subtle regulatory mechanisms.

**Fig. 1:**
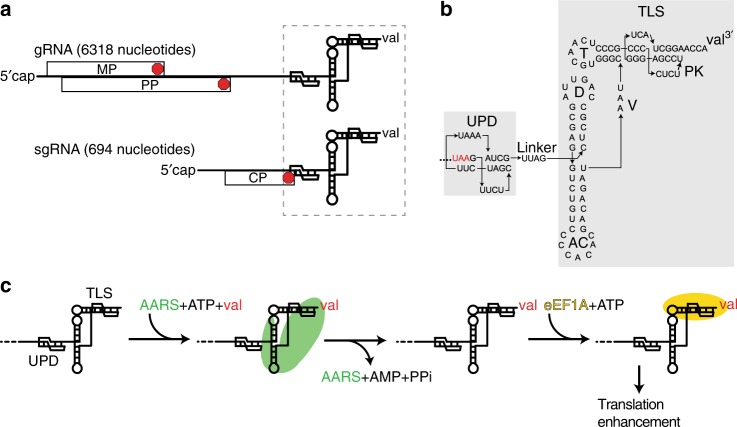
Location, structure, and function of the TYMV 3′-UTR. **a** TYMV gRNA and sgRNA. ORFs and the 3′-UTR are labeled. MP movement protein, PP polyprotein, CP coat protein. Red: stop codons. The CP ORF is translationally silent in the gRNA. Both RNAs end with identical 3′ ends that contain the UPD + TLS domain and both are valylated (dashed box). **b** Secondary structure of the 3′-UTR. Red: CP ORF stop codon. Specific parts of the structure are labeled. **c** Schematic of TYMV TLS tRNA mimicry and translation enhancement. The 3′ end of the viral genome is aminoacylated by the host’s valine tRNA synthetase (green). This is followed by binding of eukaryotic elongation factor 1A (eEF1A, yellow) and translation enhancement by an unknown mechanism

The 3′-UTR of the species *turnip yellow mosaic virus* (TYMV) is an important model for exploring important principles of RNA structure-based translational control^8,9^. During infection, TYMV produces two RNAs: a genomic RNA (gRNA) that encodes the movement and polyproteins (MP and PP), and a subgenomic RNA (sgRNA) encoding the coat protein (CP)^8,10,11^ (Fig. 1a). The CP open reading frame (ORF) is translationally silent on the gRNA, and the sgRNA is generated from the negative-strand gRNA using the “tymobox” promoter^10–12^. Both RNAs are 5′ capped, but rather than a 3′-poly(A) tail they end in the same two-folded RNA domains separated by a short unpaired linker^9,13^ (Fig. 1b). Although the 3′-UTR is identical in the gRNA and sgRNA, its location relative to the translated ORFs differs (Fig. 1a). In the sgRNA the ORF ends in the upstream pseudoknot domain (UPD), whereas in the gRNA the ORFs end well upstream. This strategy of using the same 3′-UTR in two different contexts has potential functional consequences, as the translation machinery could interact differently with each.

The 3′-most domain of the TMYV 3′-UTR is the transfer RNA (tRNA)-like structure (TLS) that mimics tRNA to drive aminoacylation (with valine) of the viral RNAs. Aminoacylation plays multiple roles, including stabilizing the RNA and promoting binding of eukaryotic elongation factor 1A (eEF1A), which enhances the translation of viral proteins by an unknown mechanism^9,14–17^ (Fig. 1c). The TLS is also the binding site for the viral RNA-dependent RNA polymerase (RdRP) and must readily unwind to allow for negative strand synthesis^18,19^. This multifunctionality appears to rely on programmed conformational plasticity and this has been suggested to be a key feature governing TLS function^14^. The second domain within the TYMV 3′-UTR is the UPD, which was historically considered to be structurally independent of the TLS. However, deletion or mutation of the UPD decreases TLS-dependent aminoacylation and the presence of the UPD stabilizes the overall fold of the TLS, implying the existence of interdomain structural and functional coupling^9,20^. It is hypothesized that the two domains within this 3′-UTR undergo conformational changes that enable these multiple functions and may help to organize different processes during viral infection^20^.

Understanding how the two domains of the TYMV 3′-UTR relate and work together requires detailed structural information. To date, structural studies of the TYMV 3′-UTR include chemical probing^21^, a structure of the isolated 3′-end acceptor pseudoknot solved by nuclear magnetic resonance^22^, and a crystal structure of the isolated TLS domain^14^. The only direct three-dimensional structural analysis of the complete TYMV 3′-UTR is a small-angle x-ray scattering study that yielded low-resolution envelopes of its global structure^20^, but an atomic-resolution structure of the intact 3′-UTR that could reveal the details of interdomain communication had not been previously solved.

The TYMV 3′-UTR’s features make it a useful model system for understanding many aspects of RNA structure-based regulation, including how RNA conformational changes, multi-domain interactions, and the context of folded RNAs combine to regulate function. We used biochemistry, functional assays coupled with mutagenesis, biophysics, and structural approaches to explore this RNA. We discovered that the two-domain architecture allows a mechanism of regulation that depends on the context of the 3′-UTR relative to the ORF. Specifically, the UPD acts as a structure-based ribosome sensor, communicating its conformational status to the TLS to alter the efficiency of downstream functions. By solving the structure of the entire 3′-UTR by x-ray crystallography, we revealed the nature of the communication conduit between the two domains within the higher-order RNA architecture. Overall, these results provide an illustrative model of context-dependent RNA structure-based regulation of function that uses multiple domains, conformational changes, and interdomain communication.

## Results

### The TYMV 3′-UTR context affects translation

**Fig. 2:**
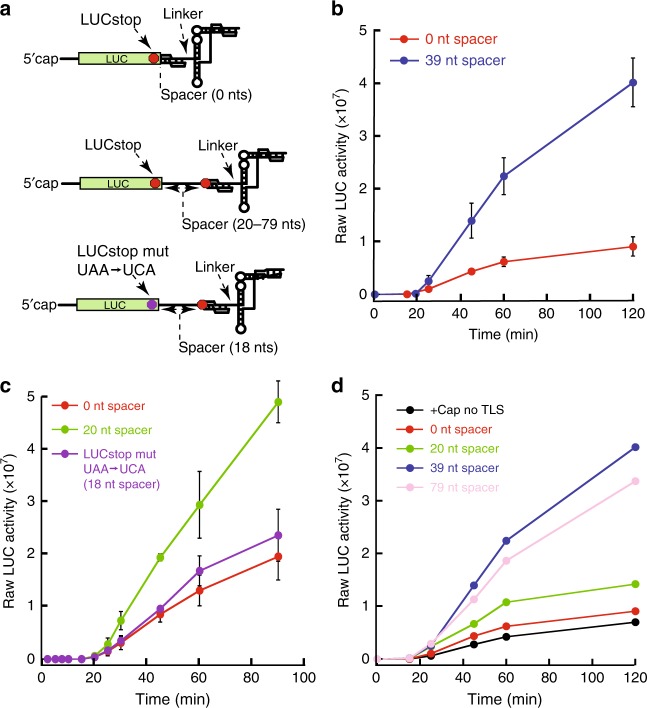
TYMV 3′-UTR context alters the rate of translation. **a** Luciferase (LUC) reporter design. Elements that were altered are indicated. The top reporter represents the 0 nt spacer, the middle reporter represents different spacer nucleotide lengths, and the bottom reporter represents the LUC stop codon mutant reporter (indicated by the magenta symbol). The red symbol represents the LUC stop codon and the stop codon that is inside the UPD. Reporters were capped unless indicated. **b**–**d** In vitro translation assays with: **b** two different spacers between the two stop codons; **c** two spacer lengths and the upstream stop codon mutated; **d** four spacer lengths with data showing the mean translation levels for *n* ≥ 3 replicates. Absolute differences in LUC activity between panels are due to different amounts of lysate used. Error bars represent one s.e.m. of *n* ≥ 3 replicates. Sequence details in Supplementary Fig. 2

We first recapitulated 5′ cap-dependent translation enhancement by the TYMV 3′-UTR using luciferase-encoding reporter RNAs in cell-free translationally competent wheat germ extracts (WGEs). In this system, the presence of a 5′ cap dramatically increased the rate of translation initiation, and the presence of the TYMV 3′-UTR further enhanced translation of the upstream reporter ORF (Supplementary Fig. 1a, b). Using this assay, we first determined the functional effect of the location of the 3′-UTR relative to the associated ORF using constructs with variable length spacers between the luciferase stop codon and the UPD (Fig. 2a). With a 39-nucleotide-long spacer, translation was enhanced relative to a reporter with the stop codon placed as the first three nucleotides in the UPD (0-nucleotide-long) as in the sgRNA (Fig. 2b; all mutants used in this study are shown in Supplementary Fig. 2). Using a reporter with an 18-nucleotide-long spacer, we mutated the upstream stop codon to allow ribosomes to progress to the stop codon in the UPD; translation decreased to the level of the 0-nucleotide-long spacer reporter (Fig. 2c). Lengthening the spacer incrementally from 0 to 39 nucleotides resulted in increased translation that was not further increased with a 79-nucleotide-long spacer (Fig. 2d). These data show that the context of the 3′-UTR matters: within a certain distance, its proximity to the ribosomal stop codon alters its ability to enhance translation.

We hypothesized that the context dependence of 3′-UTR-dependent translation enhancement is due to the physical location of terminating ribosomes relative to the UPD. Indeed, there is precedent for translating ribosomes affecting RNA structure to alter other processes^23–25^. To directly detect translation-induced structural changes in the 3′ end of these reporter RNAs, we chemically probed the sgRNA-like (0 nucleotide spacer) reporter in WGE, both in the absence (translating) and presence (non-translating) of cycloheximide (Supplementary Fig. 3). When translation occurs, the UPD and other structural features of the TYMV 3′-UTR have altered chemical probing signatures relative to the non-translating control. These data suggest that arrival of the ribosome induces unfolding of the UPD and this causes conformational changes within the TLS domain. Combining these observations with the functional data described above leads to a model explaining different translation enhancement levels depending on the context of the 3′-UTR relative to the stop codon of the upstream ORF. Specifically, if the stop codon is within the UPD (as in the sgRNA), ~16 nucleotides of downstream RNA is within the ribosomal footprint and this unfolds the UPD. If the stop codon is well upstream of the UPD (as in the gRNA), the UPD structure remains folded. Because the TLS domain is outside the ribosomal footprint, it should not be unfolded with either stop codon location, but we hypothesized that it senses the status of the UPD to affect downstream functional outputs. This model requires that higher-order RNA architecture couples the two domains in the 3′-UTR.

### Two RNA domains are structurally and functionally coupled

**Fig. 3:**
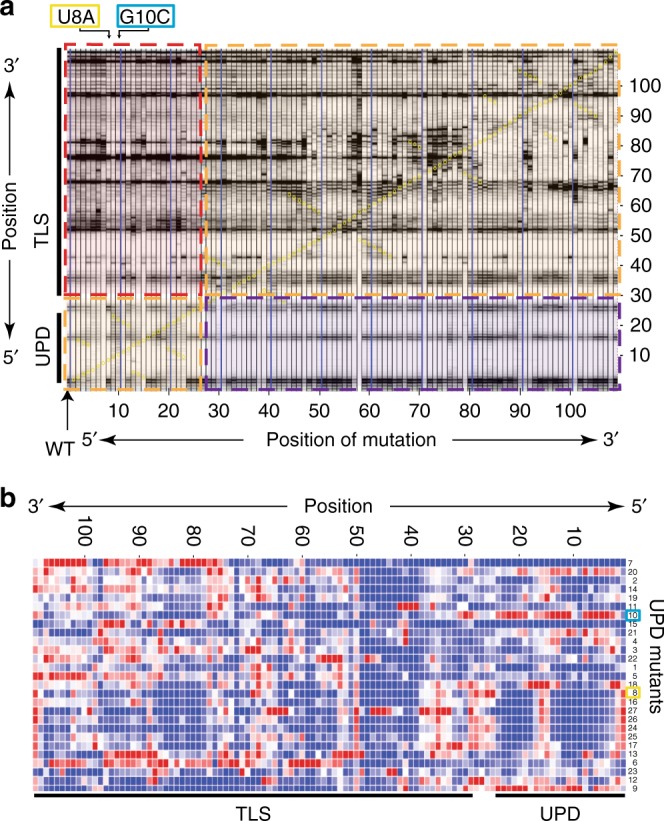
Structural coupling between RNA domains. **a** MCM with NMIA. *X*-axis: mutation position. *Y*-axis: sequence position. Domains indicated. Orange: modification patterns in a domain due to mutations within the same domain. Red and magenta: changes in one domain due to mutations in the other domain. **b** Hierarchical clustering of the difference of each mutant from the wild-type RNA (Supplementary Fig. 4). Blue: less reactive. Red: more reactive. Yellow and cyan: mutants U8A and G10C, respectively

**Fig. 4:**
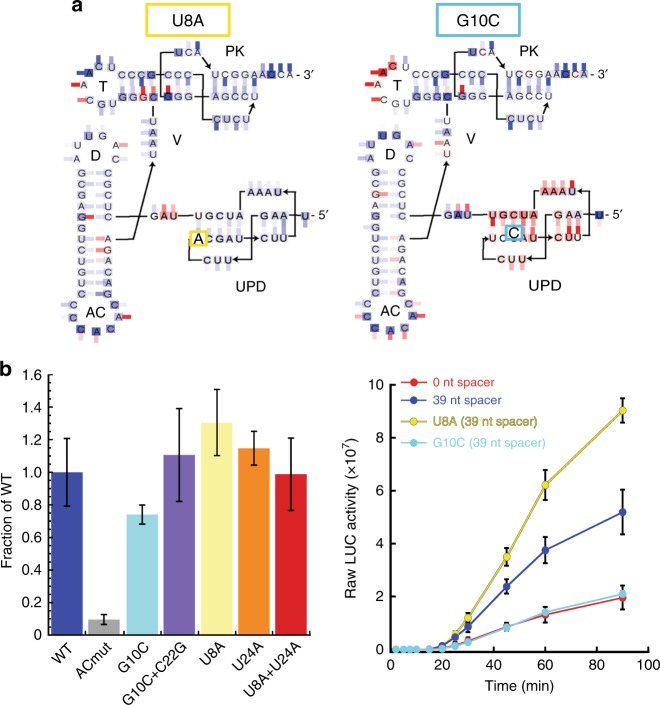
Functional coupling between RNA domains. **a** Secondary structures of the U8A and G10C mutants, colored to reflect the difference in chemical probing between mutant and WT. The new secondary structure is derived from the x-ray crystal structure (see refs. ^9,36^ for previously predicted secondary structures). Base shading represents NMIA probing and tick marks represent DMS or CMCT probing, colored as in Fig. 3b. **b** Left: in vitro valylation assay comparing mutants U8A, U24A, the double mutant U8A + U24A, G10C, and the compensatory mutant G10C + C22G to WT and a mutant with altered anticodon loop (ACmut) to inhibit valylation. Right: Translation assay comparing the U8A and G10C mutants to WT RNA reporters used to mimic the gRNA (blue) and sgRNA (red). Error bars represent one s.e.m. of *n*≥ 3 replicates

To test the first parts of this model, we investigated TLS and UPD structural coupling within the global architecture of the 3′-UTR using multi-dimensional chemical mapping (MCM) applied to the entire 109-nucleotide-long 3′-UTR RNA^26–29^, comprehensively assessing the effect of individually mutating every nucleotide on the chemical reactivity of the molecule (Fig. 3a and Supplementary Fig. 4). Although removing the entire UPD had been observed to affect TLS structure and function^9,20^, our goal was to directly assess structural coupling in a more precise way. Specifically, we wanted to identify point mutants in the UPD that alter the conformation of the TLS and that would allow us to either (1) map the location and nature of direct contacts between the domains or (2) observe how changes in the conformational stability of one domain affects the structural coupling. Several such mutants were identified, including mutations in the UPD and the interdomain linker (positions U1 to G27) that alter the TLS conformation: some destabilized the TLS while others stabilized it. Hierarchical clustering analysis identified two point mutations within the UPD as representative of these effects (Fig. 3b and Supplementary Fig. 5). Specifically, mutating nucleotide U8 to an A resulted in a chemical probing pattern that overall was consistent with a general stabilization of the native structure of the TLS. In contrast, mutation of G10 (predicted to base-pair within one stem of the UPD) to a C resulted in a chemical probing pattern that suggested a destabilization of both the UPD and the TLS (Fig. 4a). These two mutants were selected for further study as they are proximal in sequence, but their mutations initially appeared to have different conformational effects; in addition, one was predicted to be unpaired, while the other was predicted to be base-paired within a stem in the UPD, making them useful for comparison.

The chemical probing suggested that mutations U8A and G10C affect the conformation of the 3′-UTR in opposing ways; to better understand the nature of the induced effects, we characterized them biophysically, using sedimentation velocity analytical ultracentrifugation with in vitro-transcribed RNAs containing the entire 3′-UTR. Comparing the measured sedimentation and diffusion coefficients of wild-type (WT) RNA with and without magnesium shows a change in the values consistent with the RNA adopting its native folded structure in the presence of the metal ions, agreeing with previous studies^30^ (Table 1). Mutant U8A’s measured parameters are virtually identical to those of WT, indicating that the overall conformation has not changed with the introduction of the mutation. In contrast, the G10C mutation causes a change in the measured parameters, with small but measurable changes in the sedimentation and diffusion coefficients and corresponding increases in the resultant *f*/*f*_0_ and hydrodynamic radius values. The small changes are consistent with the G10C mutation altering or destabilizing the conformation of the UPD but not causing the RNA to fully unfold. We further tested this interpretation using thermal denaturation monitored by ultraviolet (UV) absorbance to detect mutant-induced changes in structural stability (Supplementary Fig. 6). WT and mutant U8A have very similar profiles, with U8A showing perhaps a slight stabilization in the overall fold. In contrast, mutant G10C began unfolding at lower temperatures than did WT and had a different profile in the lower-temperature region of the curve where tertiary structures would be expected to unfold (~30–50 °C). These results are consistent with a general destabilization of the global architecture of the 3′-UTR with the G10C mutation and a subtle change in conformation and thermodynamic stability rather than a complete unfolding event.

**Table 1:** Sedimentation velocity analytical ultracentrifugation measurements

RNA species	EDTA or MgCl_2_	Measured values		Calculated values	
		*S*_20,*w*_ (Svedbergs)	*D*_20,*w*_ (x10^7^ cm^2^/s)	*f*/*f*_0_	*R*_H_ (Å)
WT	1 mM EDTA	3.67 ± 0.02	5.92 ± 0.03	1.92	36.7
WT	2 mM MgCl_2_	4.86 ± 0.01	7.02 ± 0.33	1.49	28.5
Mut U8A	2 mM MgCl_2_	4.86 ± 0.01	7.00 ± 0.32	1.49	28.5
Mut G10C	2 mM MgCl_2_	4.72 ± 0.02	7.89 ± 0.26	1.53	29.3

We determined if the different chemical probing patterns and biophysical behavior of mutants U8A and G10C correlate with changes in function. Using in vitro valylation assays with purified valine synthetase enzyme and the entire 3′-UTR, we observed that U8A is aminoacylated at levels at least as well as WT, while G10C has decreased aminoacylation levels (Fig. 4b). Mutant G10C’s ability to be aminoacylated did not drop to the level of a mutant in which the anticodon loop was mutated to prevent recognition by the synthetase, again consistent with the idea that the mutation destabilizes but does not fully disrupt folding of the TLS. Finally, we tested the ability of mutants U8A and G10C to enhance translation of an upstream reporter, using constructs with a 39-nucleotide-long spacer to decouple the effects of the mutants from any ribosome-induced effects (Fig. 4b). Mutant U8A enhances translation to above WT, while mutant G10C’s translation activity was decreased to a level similar to that of a reporter with the stop codon in the UPD.

### The structure reveals no direct interdomain contacts

**Fig. 5:**
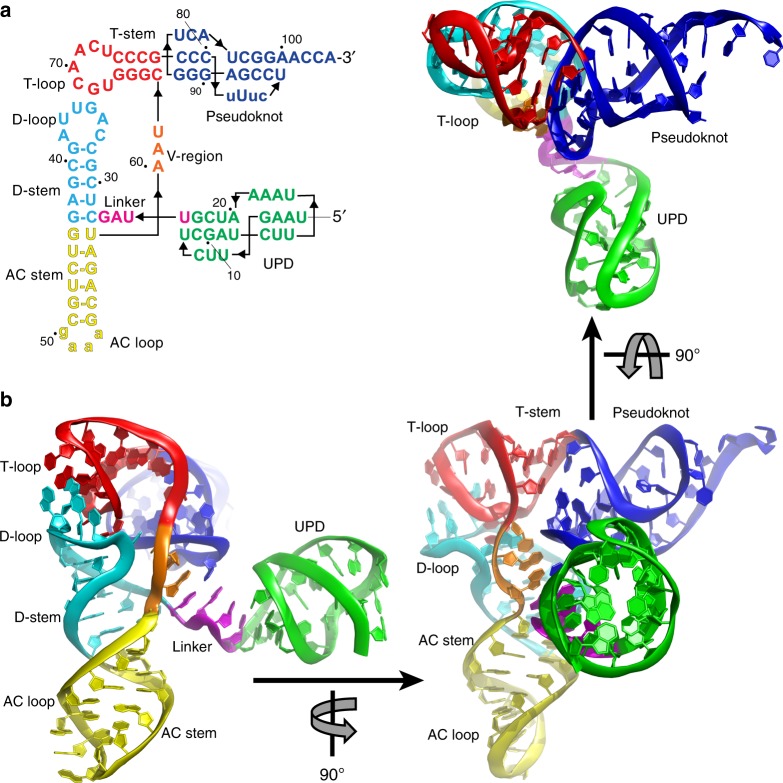
TYMV 3′-UTR structure. **a** Secondary structure of the crystallized RNA. Lowercase: sequences altered for crystallization; this includes nucleotides that enhance aminoacylation in vitro^71^. **b** Ribbon representation of the structure, colors match **a**

The data presented above show that the UPD and TLS are structurally and functionally coupled and this architecture provides a means to regulate translation, but high-resolution structural information that explains these phenomena was lacking. Specifically, although the high-resolution structure of the isolated TLS was previously solved^14^, and low-resolution reconstruction of the shape of the 3′-UTR had been generated by small-angle X-ray scattering (SAXS)^20^, the high-resolution structure of the UPD and of the full 3′-UTR RNA were unsolved. Therefore, we solved the structure of the entire TYMV 3′-UTR by x-ray crystallography to a resolution of 3.1 Å (Fig. 5a, Supplementary Table 1). The crystallographic asymmetric unit contains two copies of the RNA that are structurally almost identical; in both, all nucleotides except the 5′-terminal U and 3′-terminal CCA sequences had density of high enough quality that they could be built (Supplementary Fig. 7a).

**Fig. 6:**
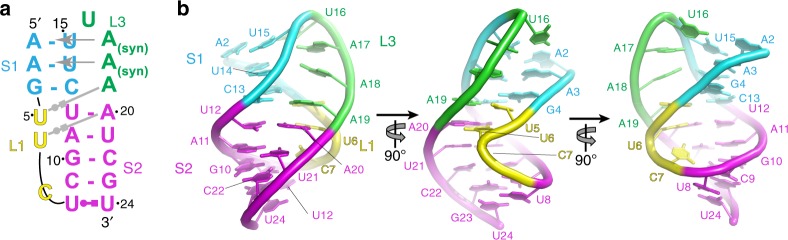
Structure of the UPD. **a** Secondary structure diagram of the UPD, colored by element (colors do not match Fig. 5) and labeled. Grey arrows indicate where bases interact with the minor grooves and non-canonical base pairs are shown. **b** Structure of the UPD in ribbon representation, colored to match **a**. Nucleotides are numbered

The crystal structure reveals the conformation of each of the two domains and their relationship to one another. Overall, the TLS domain maintained the tRNA L-shape fold, matching the isolated TLS structure^14^ (Supplementary Fig. 7d). Thus, the presence of the UPD does not alter the conformation of the TLS and the interdomain communication is not through dramatic structural changes in the TLS. The UPD forms a compact H-type pseudoknot (Fig. 6)^31^. Consistent with this type of pseudoknot, Loop 1 (L1, nucleotides 5–7) lies in the major groove of Stem 2 (S2), with U5 and U6 pairing with the Hoogsteen edges of A19 and A20, respectively (the latter forms a base triple). The A bases of loop 3 (L3, nucleotides 16–19) lie in the minor groove of Stem 1 (S1), with two of them (A17 and A18) in a syn conformation. S1 and S2 do not coaxially stack on one another, but are offset to allow L3 to stack on a strand of S2 and L1 on a strand of S1. In addition, a previously unpredicted non-canonical base pair forms between U8 and U24; this pair stacks on Stem 2. Given the observed structural coupling between the UPD and TLS, we expected them to be in direct contact. Surprisingly, the UPD assumes an angle roughly perpendicular to the TLS’ helices with no direct contact between the two domains (Fig. 5b). Also, the position of the UPD relative to the TLS is distinct from that of long variable loop extensions in tRNAs (Supplementary Fig. 7e); thus, the architecture is not a direct mimic of these types of tRNAs. The unexpected position of the UPD relative to the TLS could be due to crystal packing, but several lines of evidence argue against this. First, there are two RNA molecules in the asymmetric unit with different crystal contacts but identical UPD positions; this is highly unlikely to occur if crystal packing dictates the interdomain orientation (Supplementary Fig. 7b and c). Second, we compared previously collected experimental SAXS data of the full 3′-UTR with scattering curves predicted using models of the UPD and TLS at different relative angles (Supplementary Fig. 8)^20^. The crystallized configuration agrees best with the experimental SAXS data. This interpretation does not preclude movement of the domains relative to one another, but it strongly suggests the crystal structure reflects the average, thermodynamically favored position of the domains relative to one another in solution. Additional evidence is found in the structure of the linker that connects the two domains, described below.

### An unusually structured linker connects the two domains

**Fig. 7:**
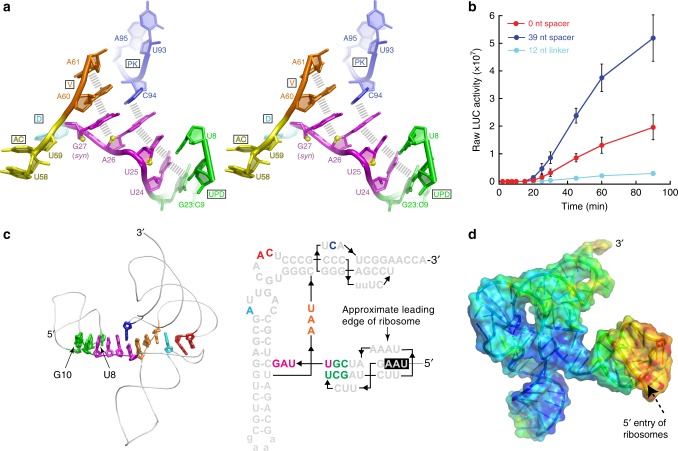
Interdomain communication and the spine structure. **a** Stereoview of the linker region and surrounding structure. Yellow spheres indicate the O4′ atom to highlight the double inversion. Grey hashes: stacking. The Z-DNA-like motif is found in the *syn*-G27 and A26 step. **b** In vitro translation assays of a reporter with the interdomain linker length increased to 12 nucleotides. Data from two different spacers is included for comparison. Error bars represent one s.e.m. of *n* ≥ 3 replicates. **c** Left: A spine of continuously stacked bases extends from the UPD to the T-loop. Nucleotides in the spine are shown in stick representation (colored to match Fig. 5, G10C and U8A positions are shown). Right: Secondary structure of the TLS + UPD, with nucleotides visible to the left highlighted and colored to match. The stop codon in the UPD is boxed in black. The location of the leading edge of the ribosome when the stop codon is in the A site is shown. **d** Surface representation of the structure colored by relative crystallographic *B*-factor, with red highest and blue lowest

The lack of direct TLS-UPD packing suggests that interdomain communication occurs through the linker, an idea supported by its location, structure, and previously reported stabilizing effects^20,30^. Although nucleotides in the linker are not base paired, they form a well-defined structure containing several unusual features. The linker contains a Z-DNA-like element (in this case, a single Z-step) and an S-turn motif, with the sugar-phosphate backbone going through two complete inversions in three nucleotides^32^ (Fig. 7a). Z-DNA-like elements are motifs in which the RNA locally adopts a left-handed helical conformation, with a ribose O4′ atom (in this case, A26) stacking with the next base that is in a *syn* conformation (here, G27). Z-DNA-like elements form precise turns at critical locations in diverse RNA structures and have been found in some of the most stable RNA structures known^33^. This Z-step conformation was observed in the previous structure of the isolated TLS domain; hence, it is an authentic part of the linker conformation^14^. S-turns are common but the version seen here is unusual, being unpaired when most such turns are found in paired regions such as the common E-loop motif^34,35^. The linker structure appears to be stabilized by extensive base stacking: the non-canonical U8:U24 base pair (not previously predicted^36^) in the UPD stacks with U25; A26 stacks directly between U25 and the syn-G27:C94 base pair (within the Z-DNA-like element). Communication of the structural status of the UPD to the TLS likely depends on this linker structure. To test this, we used a reporter with the stop codon 39 nucleotides upstream from the UPD, but increased the linker length to 12 nucleotides to disrupt the base stacking and the proximity of the two domains. Translation of this reporter was severely reduced (Fig. 7b).

### A spine of stacked bases functionally links the domains

The high-resolution structure reveals that the linker is part of a “spine” of continuously stacked nucleobases that extends from the UPD, through the linker, to the TLS’ V-region and the D-loop at A38, which interacts with the T-loop (Fig. 7c). This spine contains nucleotides from all of these elements, effectively linking them within the structure. In addition, the syn-G27:C94 base pair in this continuous stack links the acceptor stem pseudoknot to this 3′-UTR conduit; this base pair has been dubbed the “linchpin” as it is a key stability element in the TLS tRNA-like fold and may be important for RdRP-linked destabilization of the TLS portion of the spine^14^ (Fig. 7a, c). These stacked nucleotides are well-ordered, with low crystallographic *B*-factors relative to other parts of the structure (Fig. 7d). The term spine is thus appropriate as it likely provides both conformational stability to the entire 3′-UTR architecture and creates a communication conduit between distal structural elements.

The behavior of U8A and G10C mutants support the idea that the spine is important for linking the two structures. First, G10C is predicted to destabilize the UPD by disrupting a base pair in Stem 2, which should propagate through the linker to the TLS. We tested this idea by examining the effect of restoring the base pair with a compensatory mutation (G10C + C22G), using chemical probing and functional analysis (Supplementary Fig. 6). While mutation G10C caused a change in the chemical probing consistent with destabilization throughout the 3′-UTR and a loss of valylation efficiency relative to WT, compensatory G10C + C22G restored the WT probing pattern and valylation efficiency (Fig. 4b). Second, U8A converts the non-canonical U8:U24 pair at the UPD–linker interface to a potential A–U base pair; this could favor stacking with the linker which would propagate to the TLS. As there is not a “compensatory” mutation to be made in this case, we tested mutants that convert the U8:U24 pair (WT) to U8-A24 and to A8-A24 in addition to the U8A mutant. The chemical probing pattern of all of these mutants is very similar to WT. In valylation assays, the mutants that create an A–U pair are valylated at or slightly above WT levels, while the double mutant U8A + U24A matched WT (Fig. 4b). Thus, all of these pairings are tolerated in the structure. The results are consistent with the idea that the identity of the base pair in this location is not crucial, although an A–U pair may improve stacking interactions between the UPD and the linker.

The structural and functional data support a model that links ribosome-induced conformational changes to one domain, communication between the two domains, and regulation of translation. Specifically, we propose that within the overall 3′-UTR architecture, the folded UPD stabilizes the TLS conformation through the continuous base-stacking spine, creating a favorable substrate for aminoacylation, eEF1A binding, and translation enhancement. If a ribosome reaches the stop codon in the UPD (in the sgRNA), it will unfold the structure, which propagates through the spine to destabilize the TLS and depress translation enhancement. Consistent with this, the in-lysate chemical probing data show that during translation, there is increased reactivity of nucleotides in the same regions of the TLS that show increases with the G10C mutation and that are associated with the spine of stacked nucleotides (Fig. 4a, Supplementary Fig. 3 and 9). In addition, in line with this model of ribosome-induced unfolding, the side of the UPD that the ribosome’s leading edge encounters is less defined in the electron density (Fig. 7d), possibly indicating it is structurally unstable and primed for unfolding.

## Discussion

RNA structure-based regulation of biological processes is ubiquitous, yet many of the fundamental mechanistic strategies by which changes in RNA conformation result in regulation remain unknown. We used the 3′-UTR from the TYMV as a model to explore how the coupled stabilities of two seemingly independent RNA domains could be used to create an RNA conformation-dependent regulatory platform. We propose that one domain (the UPD) acts as a ribosome sensor, communicating information to the other domain (the TLS), which results in subtle changes in stability and subsequent changes in the ability of the 3′-UTR to enhance translation. The structure of the entire 3′-UTR shows that this interdomain communication is through a spine of continuously stacked bases that appears to create a conduit for structural and thus functional coupling.

**Fig. 8:**
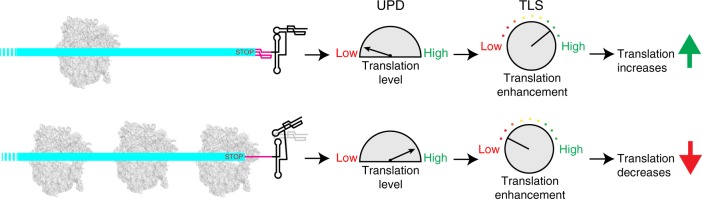
A model for ribosome-induced programmed conformational changes. Schematic cartoon of the model of translation regulation on the sgRNA. With few translating ribosomes, the UPD sensor is mostly undisturbed, the TLS is stabilized, and translation is enhanced. When translation is high (many ribosomes), the UPD spends more time in an unfolded state, resulting in destabilization of the TLS structure and decreased translation enhancement

Our studies do not directly address the virological purpose or advantage conferred by this 3′-UTR-based regulation strategy, but we propose a model. In the cell, this 3′-UTR could operate within a larger context as part of an RNA-based rheostat that tunes production of CP encoded by the sgRNA to a virologically optimal level, independent of the production of MP and PP (Fig. 8). Specifically, if ribosome density on the sgRNA is low, the UPD will be structurally unperturbed most of the time with corresponding stabilization of the TLS, resulting in increased aminoacylation and translation enhancement. The result will be to increase CP production. In contrast, if ribosome density on the sgRNA is high with corresponding rapid CP production, the UPD will be unfolded a greater percentage of the time and thus translation-enhancing effects will be depressed. This ability to directly sense and respond to the degree to which an individual mRNA is being translated would thus provide an exquisitely sensitive way to regulate translation, providing an additional layer of regulation in addition to any conferred by the action of the subgenomic promoter. Note that this form of regulation will only occur on the sgRNA where the ORF ends in the UPD and will not occur on the gRNA. Thus, differential regulation can be achieved on two RNAs derived from the same template using the same RNA structure in different contexts. Using the same 3′ end on different coding RNAs to provide specific regulation of translation has been proposed to explain the existence of some 3′ cap-independent translation enhancer (3′-CITE) RNAs found in other plant-infecting viruses^37^. In addition, previous studies of the TLS structure suggest that binding of the viral RdRP to the acceptor stem PK could alter interactions within the linker and spine to destabilize the TLS in a different way than would the ribosome interacting with the UPD^14^, a function likely important on the gRNA. Thus, our data, combined with published results, suggest that the TYMV 3′-UTR architecture creates a multi-domain “regulatory platform” capable of interacting with diverse partners in different contexts and responding in specific ways.

The structure-based mechanism of the TYMV 3′-UTR invites comparisons to riboswitches, which bind small-molecule ligands that produce programmed conformational changes to regulate gene expression^38,39^. In general, riboswitches are described as comprising a two-domain architecture: the aptamer domain binds to cognate small-molecule ligands, resulting in stabilization of a specific conformation. The aptamer domain conformation forces an associated expression platform to adopt one of two mutually exclusive conformations to affect production of a gene product involved in the metabolism of the small-ligand effector^40^. Hence, the riboswitch senses and responds to small-molecule concentrations using RNA structure. The TYMV 3′-UTR has some similar features, comprising a two-domain architecture in which a sensor domain detects the density of ribosomes on the associated ORF (according to the model of Fig. 8), and the other acts as the functional element that structurally responds to the sensor. However, unlike riboswitches, the trigger is not stabilization of a conformation by small-molecule ligands, but complete unfolding of one domain by a large macromolecular machine and subsequent subtle destabilization of the other domain. Likewise, the nature of the communication conduit between the two domains appears to be distinct from any used by riboswitches. Thus, by the strict definition we do not refer to the TYMV 3′-UTR as a riboswitch; however, it is intriguing that some similar underlying themes apply to both. This provides insight into how structured RNA elements can act as coupled sensors and effectors, using programmed conformational changes in regulatory roles.

The results presented here lend insight into how the TYMV 3′-UTR regulates its translation enhancement function, but leave open the question of how this enhancement is achieved. In other words, how does a tRNA-mimicking domain on the 3′ end enhance translation starting from the 5′ end, analogously to a poly(A) tail but likely mechanistically different? The aforementioned 3′-CITEs found in some other plant viruses bind translation initiation factors or ribosomal subunits and bring these to the 5′ end through long-range communication, aiding the assembly of the translation machinery^41^. In contrast, the TYMV 3′-UTR is not known to bind any initiation factors and there is no known mechanism for linking the viral RNA’s 3′ and 5′ ends, but the 3′-UTR has been shown to bind purified ribosomes in vitro in addition to its eEF1A-binding ability^9,14^. Overall, the mechanism of TYMV 3′-UTR-dependent translation enhancement very likely involves long-range communication between the 5′ and 3′ ends, but how this occurs and how it results in translation enhancement remains the subject of ongoing inquiry.

It is worth considering if TYMV 3′-UTR-like strategies of regulation based on programmed RNA conformational changes, structural context, and novel interdomain communication may exist in other viral RNAs or mRNAs. There is substantial variation in the 3′-UTRs of tymoviruses both in terms of their structural architectures and requirement for tRNA mimicry and valylation; not all have a domain that mimics a tRNA^42^. Hence, while some of these viruses may use the mechanism described here, others do not, and it should not be regarded as a universal mechanism. In addition, TLS elements with very different secondary structures are found in other types of plant viruses; these drive aminoacylation with histidine or tyrosine and also have been shown to enhance translation^8,43,44^. However, there is no clear evidence that they are using a two-domain architecture or mechanism of regulation similar to the TYMV 3′-UTR. Specifically, in plant viruses such as tobacco mosaic virus and brome mosaic virus the 3′-UTRs contain multiple UPDs with different arrangements relative to the TLS and the ORF compared to the TYMV^43–48^. There likely are multiple mechanisms at play that drive increased rates of protein synthesis from structured 3′-UTRs found in these viruses; however, this does not exclude that RNA interdomain communication plays an essential role in more complex viral 3′-UTRs.

The existence of elements like the TYMV 3′-UTR suggests that similar entities could be found in other viruses or even cellular mRNAs^49^ with mechanistic variations. Indeed, the 3′-UTRs of eukaryotic mRNA are often quite long, with potential for the formation of specific RNA structures and thus for the presence of regulation mechanisms based on programmed structural changes, but not necessary for tRNA mimicry. Such conformational changes could be induced by protein binding, miRNA binding, post-transcriptional modifications, or even small-molecule interactions, providing diverse and alternate ways for RNA folding and conformational dynamics to regulate gene expression.

## Methods

### Generation of reporters for in vitro translation

To generate the reporter with the TYMV 3′-UTR (3′-UTR) downstream of the firefly luciferase gene, we used standard molecular cloning methods. The DNA sequence of the 3′-UTR was generated synthetically (Integrated DNA Technologies). DNA encoding the luciferase coding region was generated by PCR from a plasmid template (forward and reverse primers: 5′-GTAAAACGACGGCCAGT-3′ and 5′-CACGGCGATCTTTCCGCC-3′, respectively). The DNA fragments were assembled using Gibson assembly methods (NEB) with sequences overlapping the *Eco*RI and *Xba*I restriction sites in vector pUC19, and the TYMV 3′-UTR and luciferase coding region. Briefly, pUC19 vector (NEB) was cut with restriction enzymes *Eco*RI and *Xba*I and gel purified. Thirty-five nanograms of cut plasmid, 35 ng of luciferase-encoding DNA, and 125 ng of TYMV 3′-UTR DNA were added to 2X Gibson assembly mix and Milli-Q water. The reaction was placed at 50 °C for 50 min and then transformed into DH5-α chemically competent cells. All constructs contained the T7 RNA polymerase promoter upstream of the luciferase gene. Sequences were confirmed using the services of GeneWiz and Eton Bioscience.

### Generation of RNA for in vitro translation

Templates for in vitro transcription were amplified by PCR using either Phusion polymerase (NEB) or Pfx (Thermo) with primers M13 forward (5′-GTAAAACGACGGCCAGT-3′) and reverse primers to either exclude or include the TYMV 3′-UTR and the various plasmids described above. The reverse primers used when the TYMV 3′-UTR was included were modified to ensure precise 3′ ends (CC and CCA). Specifically, the two penultimate DNA nucleotides were modified with 2′OMe^50^ to reduce N + 1 T7 polymerase nucleotide addition. Methylated reverse primers were ordered from GE Dharmacon and IDT. PCR reactions of volume 200–500 µL were conducted, the DNA was purified using the Promega Wizard Kit, and ~1 µg of template was used in T7 in vitro transcription kits mMessage mMachine and MEGAscript (Thermo). RNAs were then purified using the Qiagen RNeasy kit and the concentration was determined using a NanoDrop spectrophotometer (Thermo) at absorbance 260 nm. The quality of the RNA was determined by 8% denaturing PAGE and imaged using ethidium bromide.

### In vitro translation

In vitro translation assays were optimized using a WGE translation system (Promega). Typically, a master mix of 60 µL was used containing 2.5 µL of amino acid mix (1:1:1 mixture of provided amino acids), 2.5 µL of 3 M KOAc, pH 7.4, 0.6 µL of 100 mM Mg(OAc)_2_, 15 µL of wheat germ extract, 33.4 µL of water, and the addition of 6.0 µL of RNA at 100 ng/µL to initiate the experiment. This assay was scaled up twofold to isolate more time points as observed in Figs. 2c, 4b, and 7b in the main text, accounting for the increase in activity observed from these assays. The translation assay was completed at room temperature with 10 µL of the above reaction removed at each time point and added to 40 µL of ice-cold 1× passive lysis buffer (Promega) and immediately frozen on dry ice. To measure luciferase activity, the reactions were thawed at room temperature and assayed using a GloMax^®^-Multi Detection system with firefly luciferase reagent (Promega). Results were analyzed using Excel (Microsoft) software with three or greater replicate measurements for each time course.

### In vitro aminoacylation

RNAs for use in in vitro valylation assays contain a 5′ leader sequence 5′-GGACACUUCCACUAA-3′, where the underlined sequence is the stop codon at the start of the UPD. In vitro-transcribed RNAs were resuspended in RNase/DNase free water to 0.5 µM, and *Thermus thermophilus* valine tRNA synthetase was resuspended to 2.0 µM in 50 mM Tris, pH 8.0, 5 mM MgCl_2_, 1 mM tris(2-carboxyethyl)phosphine HCl (TCEP), 5% (v/v) glycerol. Reactions were set up by the addition of 1 µL 10× reaction buffer (20 mM ATP, 300 mM HEPES-KOH, pH 7.5, 300 mM KCl, 50 mM MgCl_2_, 50 mM dithiothreitol (DTT)), 1 µL of 2.0 µM Valine tRNA synthetase (ValRS) enzyme, 0.5 µL ^3^H-labeled l-valine (60 Ci/mmol), 1 µL of 0.5 µM RNA (folded by a heat-cooling step), and 6.5 µL of RNase/DNase free water. Each reaction was performed in replicates of four (*n* = 4). Reaction was placed at 35 °C for ~30 min. Reactions were immediately added to an equilibrated and washed vacuum filter apparatus (GE Healthcare) in 1× wash buffer: 20 mM Bis-Tris, pH 6.5, 10 mM NaCl, 1 mM MgCl_2_, and trace xylene cyanol for visualization. The vacuum filter was assembled with (bottom to top) thick filter paper (Bio-Rad gel dryer filter paper), two layers of Hybond positively charged membrane (GE Healthcare), and 0.45 μm Tuffryn membrane filter paper (PALL Life Sciences). Each sample was then rapidly washed five times with 150 μL of 1× wash buffer. The Hybond membranes were then dried, cut, and counted by scintillation (Perkin-Elmer Tri-Carb 2910 TR). Data were analyzed using Excel (Microsoft) software and plotted using KaleidaGraph (Synergy Software).

ValRS expression and purification plasmid was obtained from Riken DNA Bank (*Thermus thermophilus* HB8, TEx18A06), which was cloned into a pET11a bacterial expression vector^51^ and expressed in BL21 DE3 cells. Six liters of LB broth were inoculated and cultures grew at 37 °C until optical density at 600 nm (OD600) was 1.5. Expression was induced by the addition of 1 mM isopropyl β-d-1-thiogalactopyranoside at 37 °C for 4 h. Cells were then centrifuged at 5000 r.p.m. for 12 min at 6 °C and pellets were collected and stored at −80 °C. Cell pellets were resuspended in 50 mM K_2_HPO_4_/KH_2_PO_4_, pH 6.0, 5 mM MgCl_2_, 10 mM DTT, and one Roche protease inhibitor tablet (EDTA-free) in a total volume of 50 mL. The resuspension was sonicated for 2 min (20 s on, 40 s off) and centrifuged at 30,000 × *g* for 30 min at 4 °C. The supernatant was collected and heated to 70 °C for 30 min and centrifuged at 30,000 × *g* for 30 min at 4 °C. Supernatant was collected and added to 20 mL of Toyopearl Buffer A and loaded onto an equilibrated Toyopearl-Butyl column in Toyopearl Buffer A: 50 mM K_2_HPO_4_/KH_2_PO_4_, pH 6.0, 0.8 M ammonium sulfate, 5 mM MgCl_2_, and 1 mM DTT. Fractions were collected using an FPLC system (GE Healthcare) by eluting samples using Toyopearl Buffer B: 50 mM K_2_HPO_4_/KH_2_PO_4_, pH 6.0, 5 mM MgCl_2_, and 1 mM DTT. Eluted fractions were analyzed by sodium dodecyl sulfate-polyacrylamide gel electrophoresis (SDS-PAGE) and dialyzed into 50 mM Tris, pH 7.2, 5 mM MgCl_2_, and 1 mM DTT at 4 °C overnight. The dialyzed sample was purified using a Hi-TRAP Q ion exchange column (GE Healthcare) with the column equilibrated in Q Buffer A: 50 mM Tris, pH 7.2, 5 mM MgCl_2_, and 1 mM DTT. The sample was eluted using Q Buffer B: 50 mM Tris, pH 7.2, 5 mM MgCl_2_, 1 M NaCl, and 1 mM DTT. Eluted fractions were analyzed by SDS-PAGE and dialyzed into 50 mM Tris, pH 8.0, 5 mM MgCl_2_, 1 mM TCEP, and 5% glycerol at 4 °C overnight. Dialyzed sample was collected, the concentration was determined using a NanoDrop spectrophotometer (Thermo), and the sample was stored at −80 °C.

### Chemical probing and clustering analysis

DNA templates to generate RNA for one- and two-dimensional chemical mapping experiments were generated by a tiling PCR strategy using the Primerize work flow (https://primerize.stanford.edu/^52,53^). Primers and primer plates to generate the WT DNA template and each of the 109 TYMV 3′-UTR point mutations were ordered from IDT and resuspended in water to a concentration of 100 µM. PCR was completed using Phusion polymerase (NEB). DNA templates were purified using Agencourt AMPure XP magnetic beads (Beckman) and resuspended in sterile water. Purified DNA templates were then in vitro transcribed using T7 RNA polymerase (NEB) and RNAs were purified using magnetic beads from a Thermo Poly(A)Purist™ MAG Kit. DNA and RNA concentrations were determined using a NanoDrop spectrophotometer (Thermo) and quality was checked by agarose gel electrophoresis.

Chemical mapping procedures were carried out as in ref ^27^. Briefly, 1.2 pmoles of RNA was re-folded and equilibrated to room temperature before adding chemical agents. In separate reactions RNA was probed using 5 μL of 12 mg/mL *N*-methylisatoic anhydride (NMIA), 0.5% dimethyl sulfate (DMS), 42 mg/mL *N*-cyclohexyl-*N*′-(2-morpholinoethyl)carbodiimide metho-*p*-toluenesulfonate (CMCT) or 2% glyoxal, and incubated at room temperature for 15–30 min. Reactions were quenched using either 2-mercaptoethanol or 2-(*N*-morpholino)ethanesulfonic acid sodium salt (MES) pH 6.0. Chemically modified RNAs were isolated using a Poly(A)Purist™ MAG Kit (Thermo) and reverse transcribed using SuperScript III reverse transcriptase (Thermo) at 42–45 °C for 40–60 min using a fluorescently labeled primer (IDT): 5′-/5-6FAM/AAAAAAAAAAAAAAAAAAAAGTTGTTGTTGTTGTTTCTTT-3′.

Labeled DNA products were eluted in HiDi formamide spiked with Gene Scan ROX 350 size standard (Thermo). Samples were run on an Applied Biosystems 3500 XL capillary electrophoresis system and the data were analyzed using HiTRACE^26,54–56^ and the RNAstructure suite (Matthews Lab, https://rna.urmc.rochester.edu/RNAstructure.html) with MatLab (MathWorks). For further information on Primerize, one- and two-dimensional chemical mapping procedures see:

HiTRACE RiboKit (https://ribokit.github.io/HiTRACE/) and

Das lab website at Stanford University (https://daslab.stanford.edu/resources/).

Difference mapping and secondary structure diagram coloring was completed using MatLab and HiTRACE RiboKit: RiboPaint (https://ribokit.github.io/RiboPaint/tutorial/).

Hierarchical clustering analysis with the completely processed data from the two-dimensional chemical mapping experiments was completed using: GENE-E (https://software.broadinstitute.org/GENE-E/) and Morpheus (https://software.broadinstitute.org/morpheus/).

For each chemical modification experiment the WT data was subtracted from each mutant, generating a difference value for every mutant at each nucleotide position using either MatLab (MathWorks) or Excel (Microsoft). The data were clustered based on chemical perturbation differences from the WT RNA across the TYMV 3′-UTR (nucleotides 1–109), the TLS domain only (28–109), and the UPD with the spacer sequence 5′-UUAG-3′ (nucleotides 1–27) using the Euclidean distance and one minus Pearson's correlation metrics (data not shown) for the NMIA, DMS, and CMCT datasets.

### In vitro transcription for structure and biophysics

DNA templates encoding the desired RNA sequences were ordered as gBlocks from IDT, cloned into pUC19, and sequenced. DNA was amplified for transcription by PCR and Phusion polymerase (NEB). DNA templates to generate RNA for crystallography contained 5′- and 3′-flanking self-cleaving ribozyme sequences that were removed during purification^57^. DNA templates for SV-AUC and melting curves only contained a 5′ hammerhead ribozyme sequence and were amplified using M13F and modified reverse primers containing 2′OMe modification (see translation assay procedures). An example template that was used for biophysical analysis:

5′-**TAATACGACTCACTATAGGG**AGATCGAGAACTTACTGATGAGTCCGTGAGGACGAAACGGTACCCGGTACCGTC*TAAGTTCTCGATCTTTAAAATCGTTAGCTCGCCAGTTAGCGAGGTCTGTCCCCACACGACAGATAATCGGGTGCAACTCCCGCCCCTCTTCCGAGGGTCATCGGAACCA-*3′

The bold sequence is the T7 promoter, the underlined sequence is the hammerhead ribozyme, and the italic sequence is the 3′-UTR. Transcription reactions were conducted using 200–500 µL volume PCR reactions to generate DNA template in 2.5 or 5 mL in vitro transcriptions. Transcription reactions contained: 8 mM each NTP, 60 mM MgCl_2_, 30 mM Tris, pH 8.0, 10 mM DTT, 0.1% spermidine, 0.1% Triton X-100, and T7 RNA polymerase, as well as 1–5 µL RNasin RNase inhibitor (Promega). Transcription reactions were incubated overnight at 37 °C, and then inorganic pyrophosphates were pelleted by centrifugation. The supernatant was subjected to ethanol precipitation and RNA was purified by 8% or 10% denaturing polyacrylamide gel electrophoresis. Bands containing the desired RNA were visualized by UV shadowing and excised, and RNA was passively eluted overnight at 4 °C into ~40 mL of diethylpyrocarbonate (DEPC)-treated milli-Q filtered water (Millipore). RNA was concentrated using Amicon Ultra concentrators (Millipore). RNA quality was checked by 8% or 10% denaturing PAGE and stained with ethidium bromide for visualization.

### Sedimentation velocity analytical ultracentrifugation

In vitro-transcribed RNAs were resuspended to 2.0 absorbance at 260 nm in 5 mM sodium cacodylate buffer, pH 6.5, in DEPC-treated water and heated to 95 °C for 3–5 min, and then slow-cooled to room temperature and diluted twofold in 2× AUC buffer (50 mM sodium cacodylate, pH 6.5, either 2 mM EDTA or 2× magnesium chloride at 4 mM). The resulting 1× AUC RNA (~1.0 OD at A260) and a buffer blank, prepared identically in the absence of RNA, were then loaded in the AUC cells. AUC was performed in a Beckman XLA centrifuge using a 4-position An60Ti rotor centrifuged at 35,000 r.p.m. for 16 h at 20 °C and 180 scans were collected at absorbance 260 nm. The WT and mutant RNAs were collected in replicates of three. Scans for each replicate were analyzed using DCDT+, SEDNTRP, and SVEDBERG to determine sedimentation coefficient, diffusion coefficient, *f*/*f*_0_, and radius of hydration, using the specific volume 0.53 cm^3^/g (refs. ^58–61^, and DCDT+ v. 2.4.3, SEDNTRP 20130813 BETA, SVEDBERG v. 7.0.6). SV-AUC software tools were available at: http://www.jphilo.mailway.com/.

### RNA crystallization and diffraction data collection

RNAs for crystallization were prepared as described above. The sequence used for in vitro transcription was:

5′-GGCTATCGAATTC**TAATACGACTCACTATA**GGGAGA*AAGATCGAGAACTTACTGATGAGTCCGAGAGGACGAAACGGTACCCGGTACCGTC*taagttctcgatctttaaaatcgttagctcgccagttagcgaggtctgcGAAAgcagataatcgggtgcaactcccgccctttctccgagggtcatcggaaccaGAGGTGCTTGTATATAACCTCCACGATGGTGCACCTTGGGCAACACCACCACTCGCTTCGGCTTGTGGTGGTGGCAAATCATCTACATTAGGATCCGTATCGG-3′

The bold sequence is T7 promoter, the italicized sequence is 5′ hammerhead ribozyme, the lowercase sequence is the RNA of interest with capitalized bases indicating those that were changed for crystallographic reasons, and the underlined sequence represents a slightly modified ribozyme sequence derived from the *Chilo* iridescent virus ribozyme^62^, which was used to generate homogenous 3′ ends. The ribozyme-cleaved RNA was purified on by 10% denaturing PAGE and re-folded at 65 °C for 3 min at 2–5 mg/mL in a buffer containing 2.5 mM MgCl_2_ and 10 mM HEPES-KOH, pH 7.5, and then allowed to equilibrate to room temperature before a final concentration of 0.5 mM spermidine was added to the RNA. Crystal Screens I and II and Natrix I and II screens from Hampton Research were used in initial screens at 4 °C, 16 °C, 20 °C, and 24 °C. Crystals were optimized using custom screens. The RNAs used for the final structure determination were crystallized in 50 mM sodium cacodylate, pH 6.5, 2.5 M ammonium sulfate, 20 mM MgCl_2_, 1.0 mM spermine, and 2 mM hexamine cobalt chloride. Crystals were flash frozen in liquid nitrogen for x-ray diffraction with the addition of 20–30% glycerol in the same crystallization buffer. Diffraction data were collected at Advanced Light Source Beamline 4.2.2 using “shutterless” collection at 100°K; 180° datasets with “oscillation” images of 0.2°. Data were indexed, integrated, and scaled using X-ray diffraction and spectroscopy^63,64^. Pointless from the CCP4 suite^65^ determined the space group was I222 and Phenix^66^ was used to determine a molecular replacement solution based on these parameters and a search model consisting of residues 1–26 and 32–82 of the TYMV TLS lacking ligands (PDB ID: 4P5J)^14^. Iterative rounds of model building and refinement using COOT^67,68^ and Phenix^66^ were used to generate a complete model of the RNA. Crystal diffraction data, phasing, and refinement statistics are contained in Supplementary Table 1.

### Small-angle X-ray scattering

SAXS scattering profiles were previously collected^20^ using the program Primus and then further analyzed using Gnom and Gasbor. Projected scattering profiles were generated by using a model of the TYMV 3′-UTR PDB file merged with the PDB ID: 4P5J in the absence and presence of the UPD, in the crystallized state, or with manual movements of the UPD relative to the TLS domain. The predicted scattering data were plotted and compared to the experimental data using CRYSOL^69,70^.

### Figure preparation

Structural figures were generated using PyMol (The PyMOL Molecular Graphics System, Version 1.8 Schrödinger, LLC).

### Reporting summary

Further information on research design is available in the Nature Research Reporting Summary linked to this article.

## Electronic supplementary material

Supplementary information

Reporting Summary

Source Data

## Data Availability

All of the data in this manuscript are available upon request from the corresponding author and in the accompanying Source Data file. Structure coordinates are available as PDB accession number 6MJ0. A reporting summary for this article is available as a Supplementary Information file.
