# Supplementary material for: Ribosome-induced RNA conformational changes in a viral 3′-UTR sense and regulate translation levels
Source: Nat Commun. 2018 Nov 29;9:5074. doi: 10.1038/s41467-018-07542-x (PMC6265322; doi:10.1038/s41467-018-07542-x)
Supplement: Supplementary file 1 — Supplementary information [file 41467_2018_7542_MOESM1_ESM.pdf]

Supplementary Information

Ribosome-induced RNA conformational changes in a viral 3′UTR sense and regulate translation levels

Erik W. Hartwick[1,2], David A. Costantino[1], Andrea MacFadden[1], Jay C. Nix[3], Siqi Tian[4], Rhiju Das[4], Jeffrey S. Kieft[1,2*]

[1]Department of Biochemistry and Molecular Genetics, University of Colorado Denver School of Medicine, Aurora, Colorado, 80045, USA

[2]RNA BioScience Initiative, University of Colorado Denver School of Medicine, Aurora, Colorado, 80045, USA

[3]Molecular Biology Consortium, Advanced Light Source, Lawrence Berkeley National Laboratory, Berkeley, California, 94720, USA

[4]Department of Biochemistry, Stanford University, Stanford, California, 94305, USA

*To whom correspondence should be addressed:

Jeffrey S. Kieft
Department of Biochemistry and Molecular Genetics
University of Colorado Denver School of Medicine
Mail Stop 8101
Aurora, CO 80045

Telephone: 303-724-3257
Fax: 303-724-3215

Email: Jeffrey.Kieft@ucdenver.edu

Supplementary information contents:

Supplementary Methods
Supplementary Figure 1: TYMV 3′UTR translation enhancement in different contexts.
Supplementary Figure 2: Mutations and sequences used.
Supplementary Figure 3. Translation dependent structural perturbation to the TYMV 3′UTR.
Supplementary Figure 4: One-dimensional and two-dimensional chemical mapping.
Supplementary Figure 5: Hierarchical clustering analysis.
Supplementary Figure 6: Biophysical characterization of the 3′UTR by thermal denaturation.
Supplementary Figure 7: Electron density, crystal packing and structural comparisons.
Supplementary Figure 8: Model of the native 3′UTR and SAXS analysis of UPD position relative to the TLS.
Supplementary Figure 9. Chemical probing of mutant RNAs.
Supplementary Table 1: X-ray data collection and refinement statistics.
Supplementary References

Supplementary Methods:

Translation-dependent structural perturbation to the TYMV 3′UTR

sgRNA reporters were *in vitro* transcribed (Thermo mMessage mMachine), purified (Qiagen RNeasy) and the quality of RNA was assessed by denaturing-PAGE. The translation extracts were pre-treated with cycloheximide (CHX) when appropriate. Typically, a 60 μL translation assay was conducted as follows: 15 μL of wheat germ extract (WGE) (Promega), 2.5 μL of amino acids mixed 1:1:1, 2.5 μL of 3 M $KOAc_2$ pH 7.5, 1.2 μL of 100 mM $MgOAc_2$, 4 μL of RNA (2.5 μg total) , 0.5 μL of 50 mg/mL CHX or 100% DMSO, and water to 60 μL. The addition of RNA started the 30-minute translation assay. 10 μL of the translation mix was removed from each sample and used for luciferase assays to monitor translation of the reporter RNAs. Briefly, this 10 μL of the translation assay was added to cold 1X passive lysis buffer (Promega) and read using luminometer (Glo Max Promega) following standard procedure. The remaining 50 μL of the reaction was chemically modified using 3 μL of 22.3 mg/mL 1M7 or 3 μL of 100% DMSO for 5 min. The reaction was quenched by adding 200 μL of 0.5 M MES pH 6.0. An equal volume of phenol:chloroform:isoamyl alcohol (PCIAA) was added and the samples were vortexed and centrifuged at 4°C for 20 mins at 21000 x g. The aqueous layer was recovered, and ethanol precipitated with the addition of 2 μL of 20 mg/mL glycogen. The precipitated RNA was then resuspended in RNase H buffer: 50 mM Tris HCl pH 8.3, 75 mM KCl, 3 mM $MgCl_2$, 10 mM DTT. 1 μL of a 10 μM stock of RNase H oligo (IDT) was annealed to the RNA that was complementary to the 3′ end of the reporter RNA upstream of the UPD and TLS region. In this oligo, 'm' indicates nucleotide positions that have 2′OMe modifications in 10 positions flanking the 'GTTT' unmodified sequence to direct site specific duplex cleavage [1].

RNase H oligo: 5′-mCmUmUmGmCmGmUmCmGmAGTTTmUmCmCmGmGmUmAmAmGmA-3′

1.5 μL of RNase H enzyme (MilliporeSigma) was added and the 20 μL sample was incubated at 37°C for 45 min. 20 μL of 5 mM EDTA was added to quench the RNase H reaction and water was added to 150 μL total volume. An equal volume of PCIAA was added to extract the RNA followed by ethanol precipitation with the addition of 2 μL of 20 mg/mL glycogen. The precipitated RNA was resuspended in 22 μL of 4M betaine and 1.2 M D-trehalose solution [2]. 2.5 μL of resuspended RNA was then added to 2.5 μL of a 2X reverse transcription mixture: 1 μL of 5X first strand buffer (Thermo), 0.25 μL of 0.1 M DTT, 0.4 μL of 10 mM dNTPs (each), 0.25 μL of reverse transcription FAM labeled primer specific to the TLS 3′ end, 0.75 μL of water, and 0.1 μL of super script III enzyme (Thermo).

TLS RT Fam primer: 5′-/5-6FAM/AAAAAAAAAAAAAAAAAAAATGGTTCCGATGACCCTCGGAAGAGG-3′

The reaction was placed at 42°C for 15 min and then moved to 55°C for one hour. 5 μL of 0.4 M NaOH was added to each reaction and incubated at 85°C for 5 min followed by a 5-minute incubation on ice. 5 μL of acid quench mix (1 volume of 5M NaCl, 1 volume of 2 M HCl, 1.5 volumes of 3 M $NaOAc_2$ pH 5.2). 10 μL of magnetic bead mix was then added (for one sample: 3 μL 5 M NaCl, 1.5 μL magnetic beads (Poly(A) Purist Kit Thermo), and 5.5 μL water). The mixture was incubated at room temperature for 10 min, 85°C for 5 min, and slow cooled to room temperature. The cDNAs were separated by magnetic force for 10 min and the supernatant was discarded. Each sample was washed twice with 70% v/v ethanol, dried, and resuspended in 12 μL of HiDi-formamide (Thermo) spiked with ROX500 (Thermo) size standard (typically 2 μL of ROX500 in 598 μL of HiDi-formamide). The eluted cDNA fragments were then loaded on 8-capillary electrophoresis machine (Thermo). The fragments were then analyzed using HiTRACE with Matlab [3].The analysis was briefly described in the main methods section. Note: the methods for reverse transcription and cDNA fragment purification followed previously published protocols [4] with the changes described above.

Thermal denaturation. RNAs were *in vitro* transcribed from PCR-generated templates for the wild-type and mutant U8A and G10C RNAs. RNAs were folded and resuspended identically to the SV-AUC samples to 0.2 mg/mL RNA in 200 µL final volume with 2 mM $MgCl_2$. Circular dichroism (data not shown) and thermal denaturation (20°C - 100°C) data were collected using a JASCO J-815 CD spectrometer by monitoring absorbance at 260 nm in replicates of two and data were processed using Excel (Microsoft) and graphed using KaleidaGraph (Synergy Software).

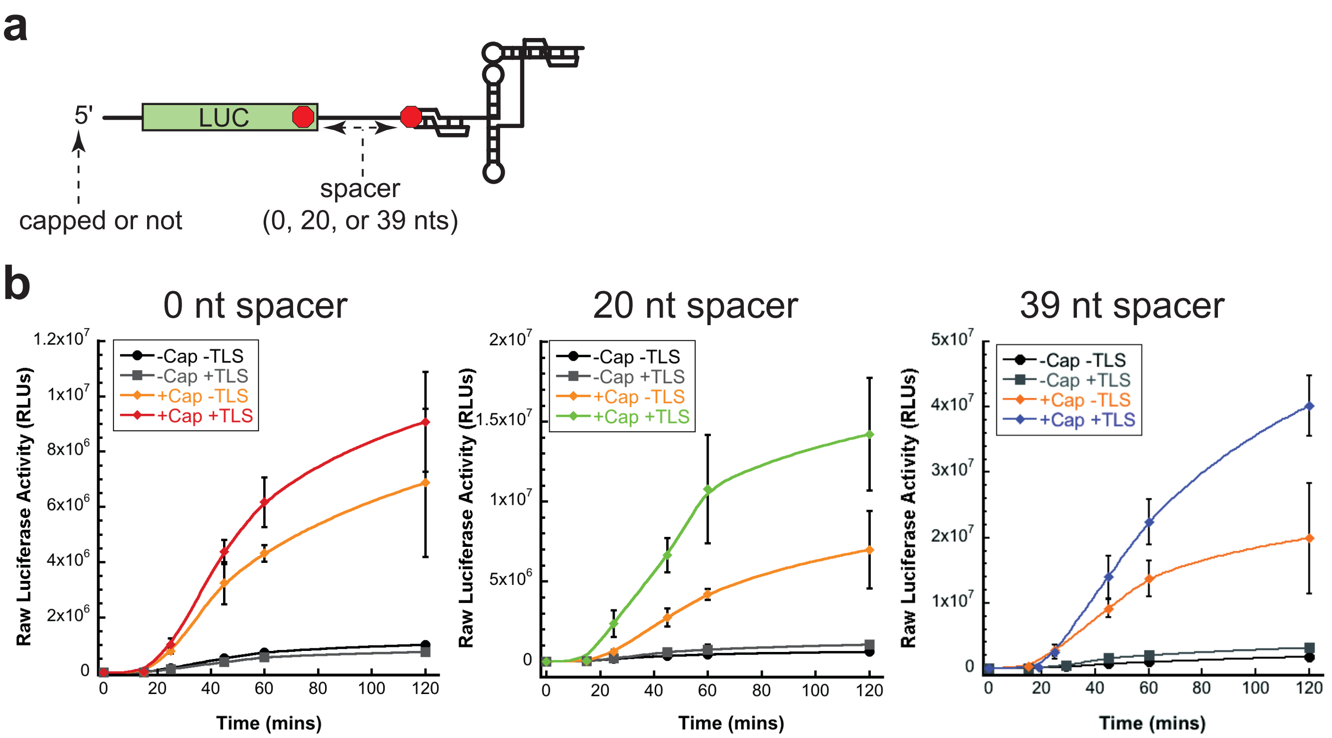

Supplementary Figure 1 TYMV 3′UTR translation enhancement in different contexts. **a,** Design of the *in vitro* translation reporters used to generate the data in panel b. The 5′ end was either capped or left uncapped, the 3′UTR was either present or completely absent, and the spacer between the luciferase reporter stop codon and the stop codon in the UPD was varied. The stop codons are represented by the red stop signs. See Supplemental Figure 2 for all sequences. **b,** *In vitro* translation assays (n ≥ 3), performed in wheat germ extract (WGE), with reporter RNAs in the absence or presence of a 5′ cap and different spacers. Error bars represent one s.e.m. of n ≥ 3 replicates.

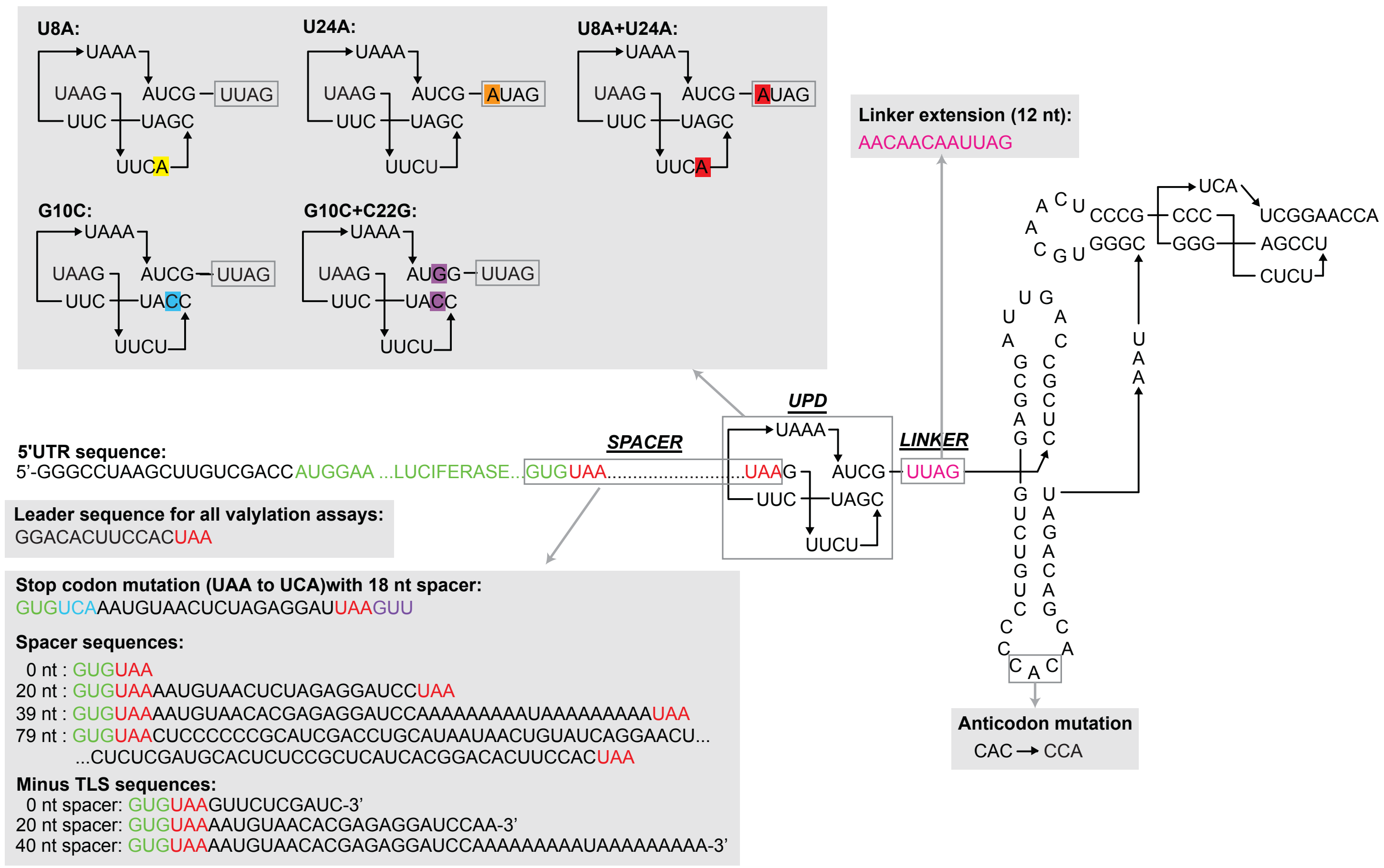

Supplementary Figure 2 Mutations and sequences used. The 5′UTR sequence is shown; the beginning of the firefly luciferase ORF (green) is the sequence used previously [5]. The last coding codon of the luciferase gene is a GUG codon, which is followed by a UAA stop codon (red). The spacer region separating the firefly ORF from the stop codon in the UPD was varied from 0 - 79 nucleotides, shown as 'spacer sequences.' The 20 nucleotide spacer is vector-derived and identical to previously published work [5]. The 18 nucleotide spacer used in conjunction with the stop codon mutation was designed based on this 20 nucleotide spacer. The 39 nucleotide spacer was also designed based on previous published work [5] where the adenosine stretches have been previously tested to avoid interaction with poly(A) binding protein [6,7]. The 79 nucleotide spacer is derived from the TYMV viral genome (GenBank: X07441.1). Reporters that lacked the UPD+TLS are shown as 'minus TLS sequences.' In the stop codon mutant, the luciferase stop codon was mutated from UAA (red) to UCA (cyan) allowing translation to continue and then terminate at the downstream stop codon in the UPD ('Stop codon mutation with 18 nucleotide spacer'). Point mutations U8A, U24A, U8A U24A double mutant, G10C, G10C+C22G compensatory mutation (yellow, orange, red, light blue, magenta, respectively) to the UPD are shown. A sequence designed to add nucleotides to the 4-nucleotide linker is the 'Linker extension'. Mutation of the anticodon loop is shown at bottom right. The 5′ leader for all of the RNA constructs used in the *in vitro* valylation assay is indicated. The 12-nucleotide sequence is derived from the WT viral RNA with the UPD stop codon position indicated in red.

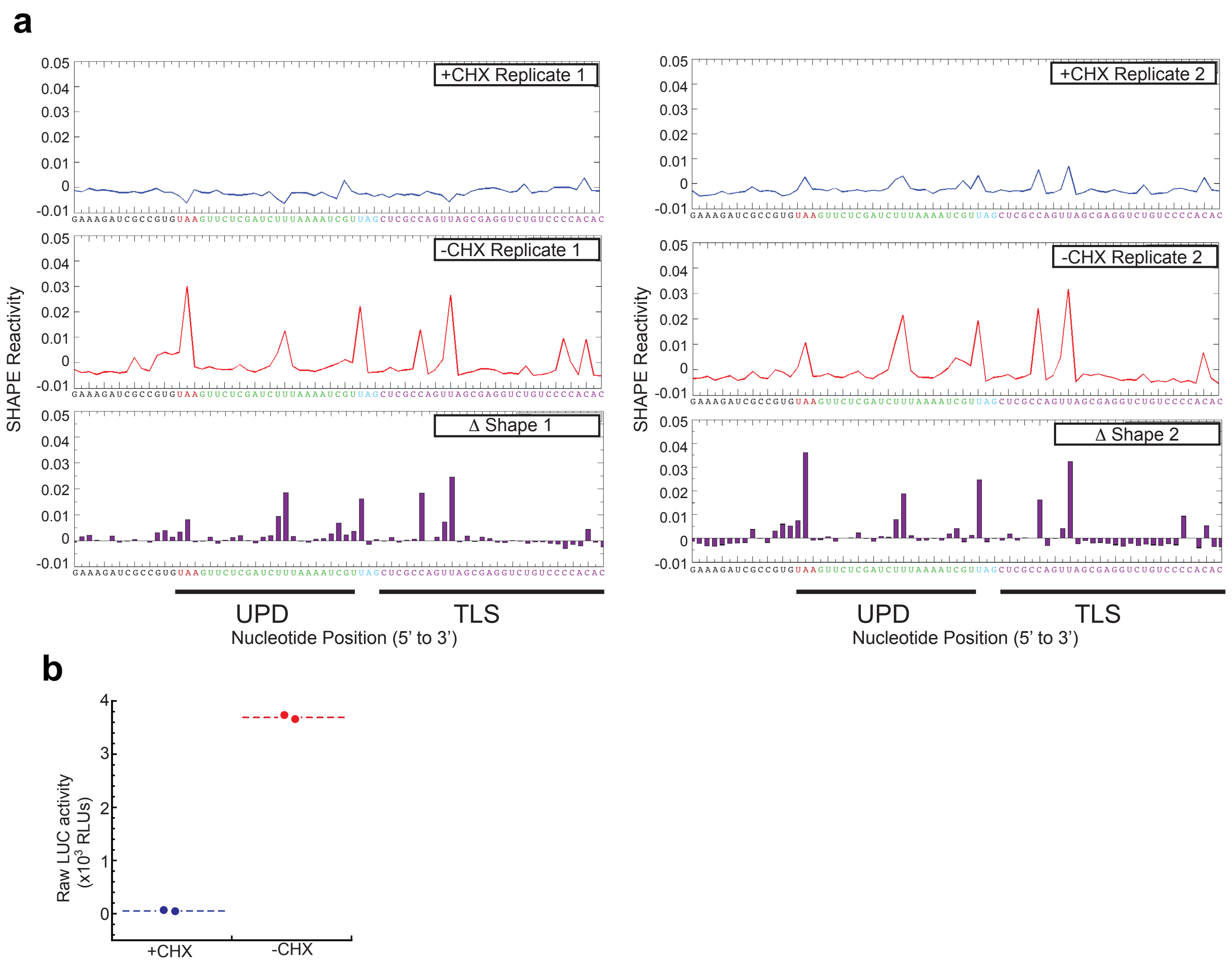

Supplementary Figure 3 Translation dependent structural perturbation to the TYMV 3′UTR. **a,** Two replicates of *in lysate* SHAPE chemical probing reactions performed using the sgRNA reporter (0 nt spacer). The blue line graphs are data from lysate pretreated with cycloheximide (+CHX). The red line graphs are data from lysates without cycloheximide (-CHX). The magenta bar graphs are the difference between the -CHX and +CHX data (Delta SHAPE). X-axis: RNA sequence with the UAA stop codon position in red, the UPD in green, the nucleotide linker in light blue, and the TLS region in magenta. Y-axis: normalized SHAPE (1M7) reactivity at each nucleotide position. Data were processed and normalized following the HiTRACE pipeline [3]. **b**, Aliquots of the lysate probing reaction were taken from the reaction prior to SHAPE modification and assayed for luciferase activity. The raw light units are plotted on the y-axis for both the + and -CHX experiments. Two replicate measurements are shown with the dashed bar representing the mean. Note that the reactions are not optimized for the amounts of reporter RNA to maximize luciferase production as in the translation assays shown elsewhere in this study.

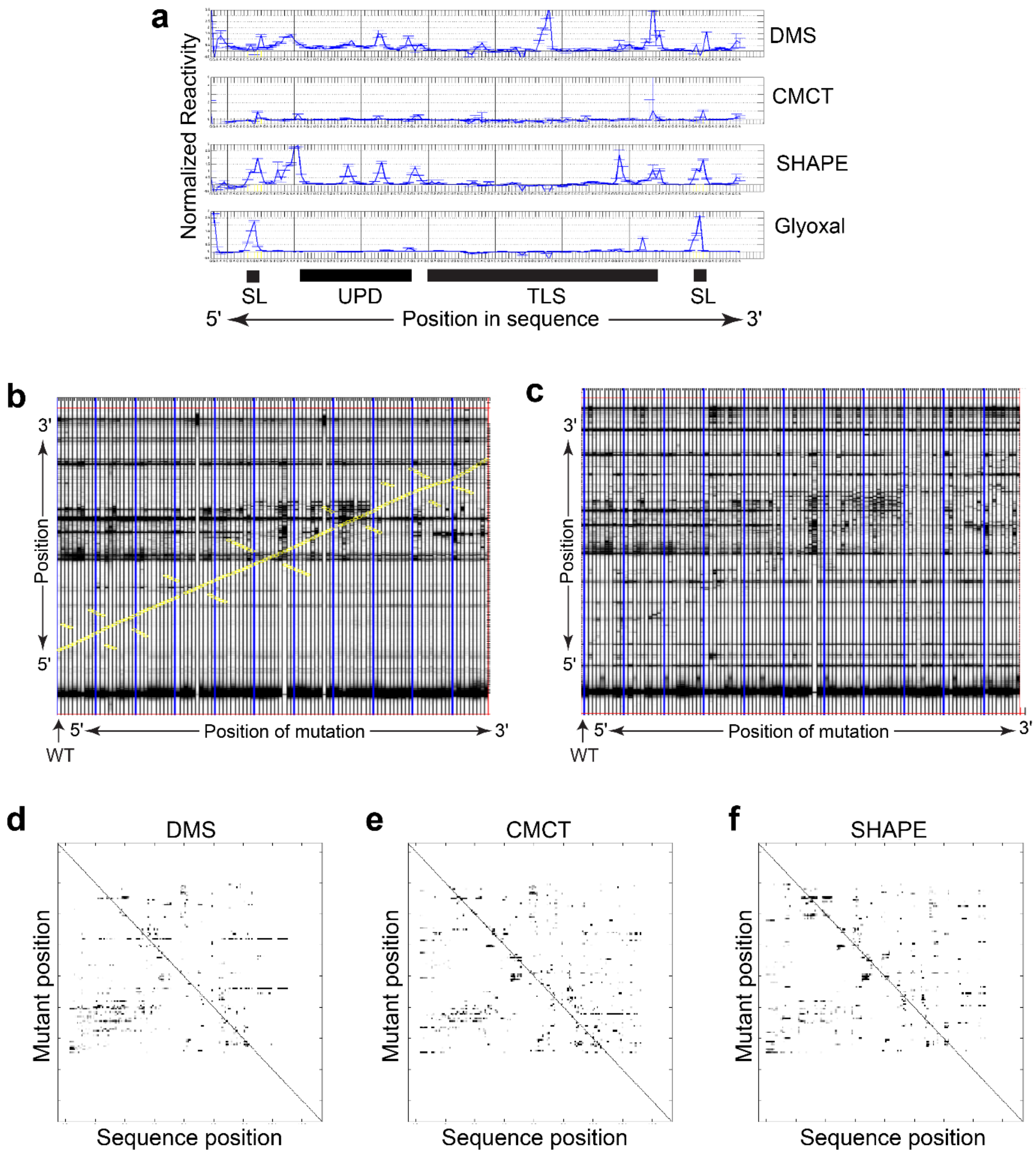

Supplementary Figure 4 One- and two-dimensional chemical mapping. **a,** Normalized one-dimensional chemical reactivity (y-axis) of the wild-type TYMV 3′UTR, probed with DMS, CMCT, NMIA (SHAPE), and glyoxal as a function of position in the RNA (x-axis). SL: Loops of the normalization hairpins used in HiTRACE analysis. The UPD and TLS regions are indicated. The 1D analysis verified that the flanking RNA sequence and construct design do not adopt alternate structures. Error bars represent one s.e.m. of $n \geq 3$ replicates. **b and c,** Multidimensional chemical mapping of the 109 nucleotide-long TYMV 3′UTR. The x-axis is mutation position and the y-axis is position in the sequence displayed from 3′ (top) to 5′ (bottom), using DMS (b) and CMCT (c). Yellow circles: Mutant position and predicted stem-loop regions on the DMS data. **d, e, and f,** Z-score analysis for the DMS (d), CMCT (e), and NMIA (SHAPE) (f) 2-dimensional chemical probing data sets, respectively. Briefly, each nucleotide across all mutants was compared and scored based on how many standard deviations from the mean a position is from the mean of all nucleotides at the same position. The x-axis is the position in sequence from the 5′ to 3′ and the y-axis is the mutation position. Black intensities represent probing positions across all mutants that stand out from the mean.

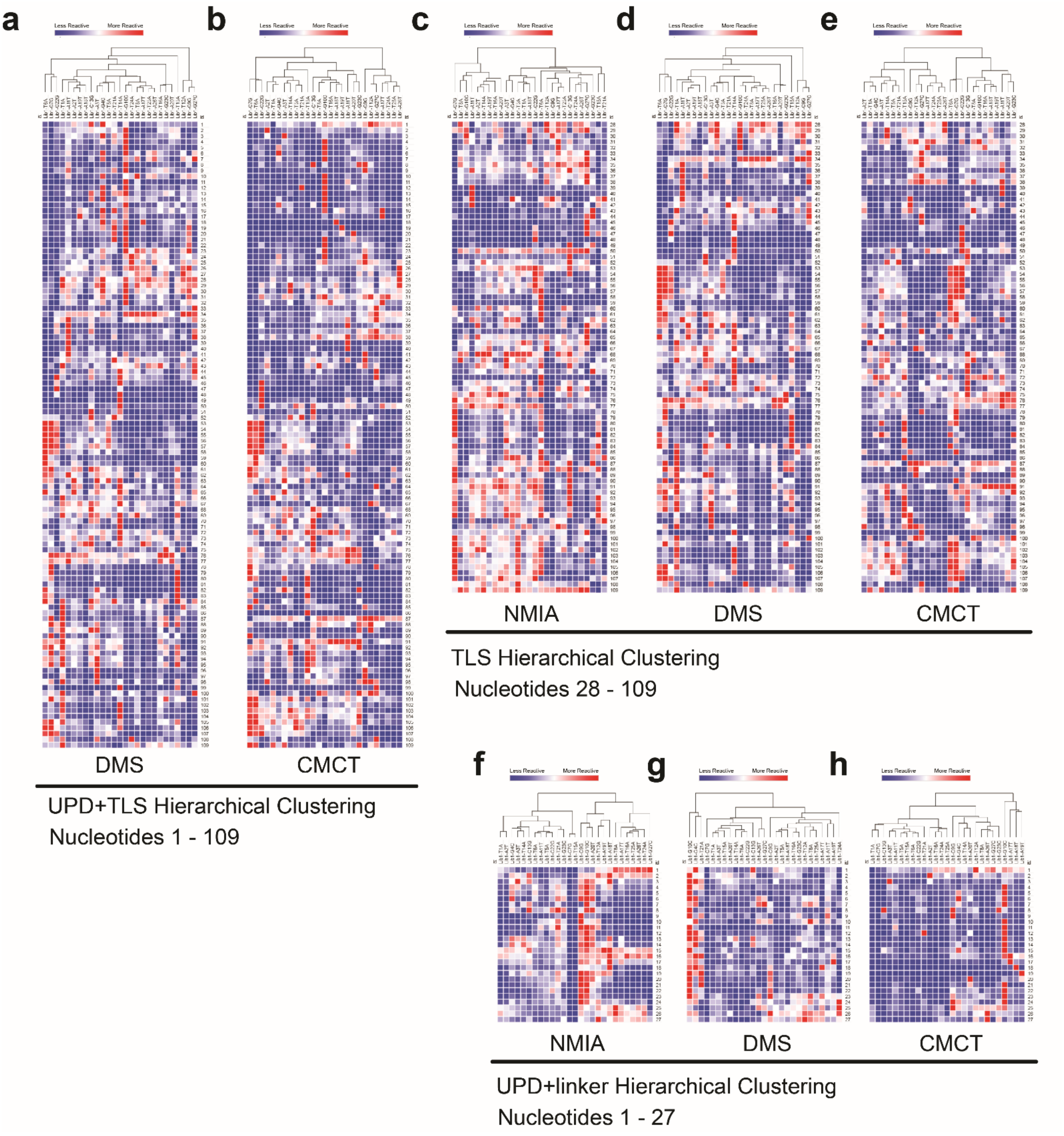

Supplementary Figure 5 Hierarchical clustering analysis. **a and b,** Difference clustering profiles for the entire TYMV 3′UTR using DMS (a) and CMCT (b) chemical probes. Mutants 1 - 27 are clustered using Euclidean distance metrics (x-axis, top) for nucleotide positions 1 - 109 (y-axis, right). Red indicates nucleotide positions that are more reactive, blue indicates positions that are less reactive**. c, d, and e,** Euclidean distance hierarchical clustering, as performed in (a) and (b), of the TLS domain only (nucleotides 28 – 109). **f, g, and h,** Hierarchical clustering of the UPD and spacer sequence only (nucleotides 1 – 27).

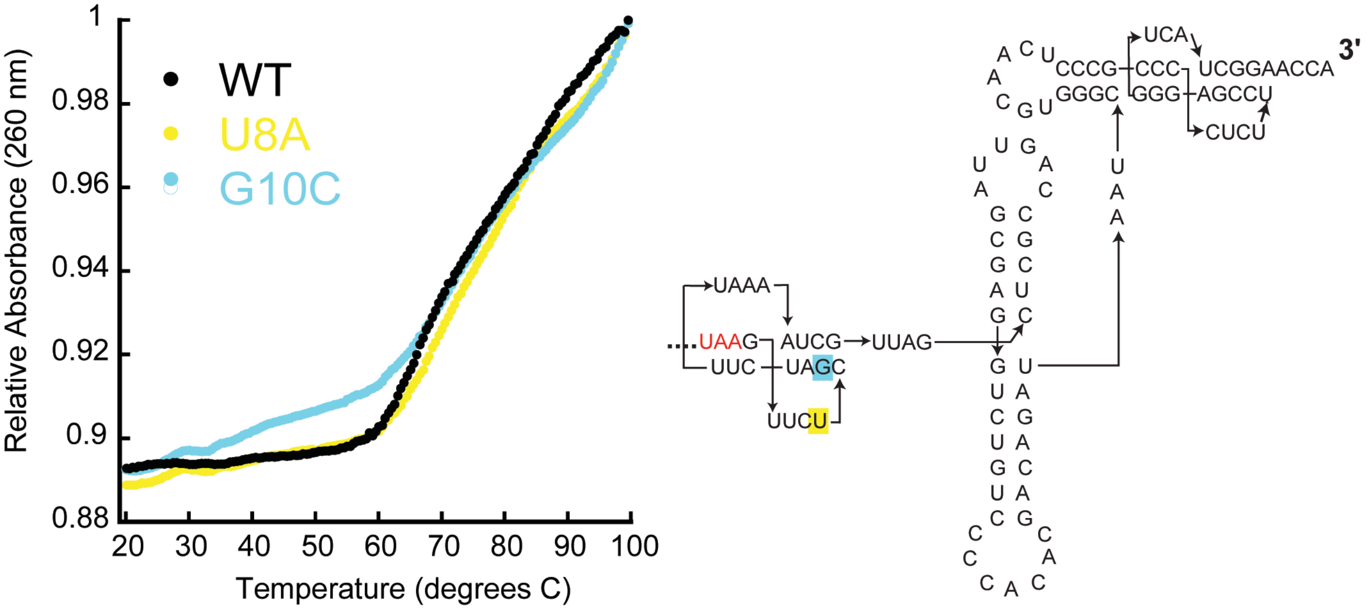

Supplementary Figure 6 Biophysical characterization of the 3′UTR by thermal denaturation. Left: Thermal denaturation curves of the wild-type RNA (black), point mutation U8A (yellow), and point mutation G10C (cyan) monitored by absorbance at 260 nm. A single replicate is shown. The plotted data are the average of two replicates. Right: Secondary structure model showing the mutant positions shaded as in the graph and the stop codon position colored in red. The RNAs used in this experiment were not aminoacylated.

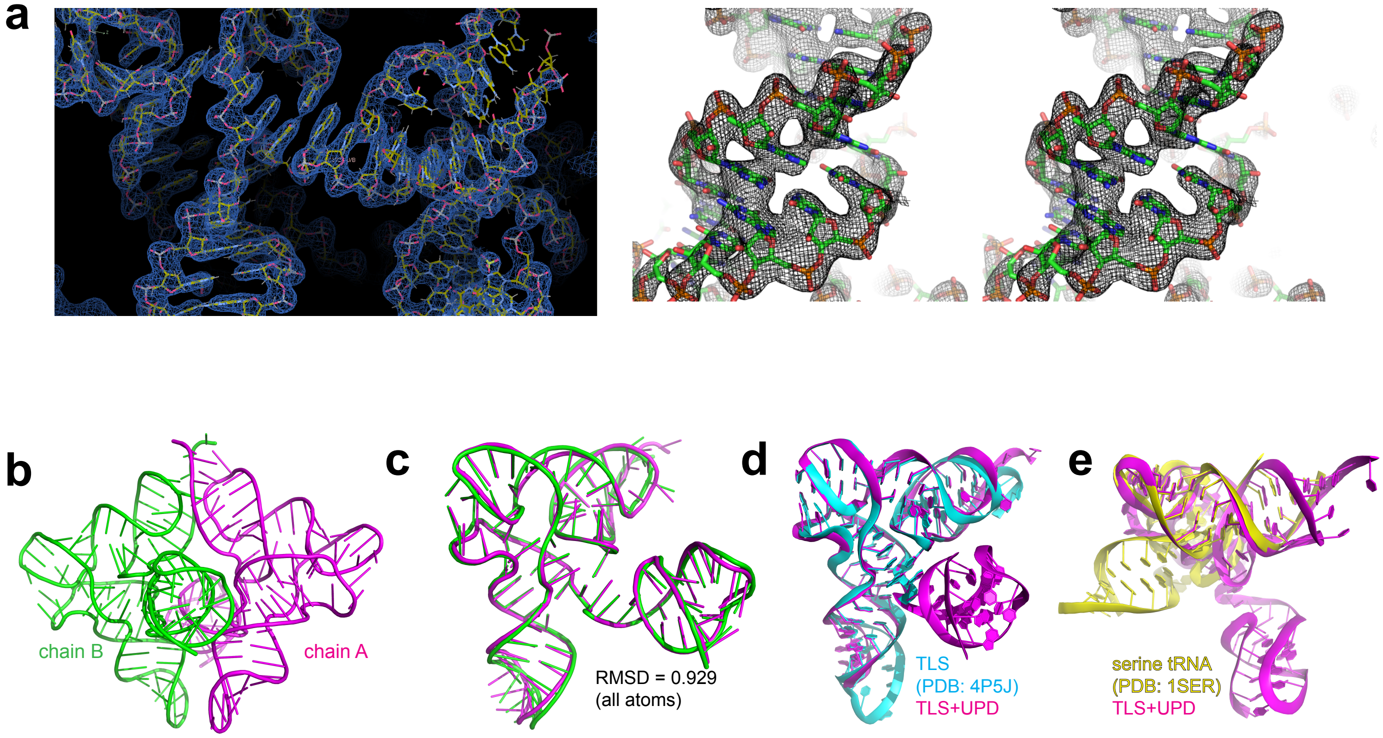

Supplementary Figure 7 Electron density, crystal packing and structural comparisons. **a, Right:** representative region of the final refined 2Fo-Fc electron density map (blue mesh) of the crystallized TYMV 3′UTR RNA (contour level ~ 0.4e/Å^3). Left: stereo image of a portion of a final simulated annealing composite omit map contoured at 2.0 sigma. **b,** The final refined structure of the crystallographic asymmetric unit containing two copies of the RNA (green and magenta). **c,** Both copies in the crystallographic asymmetric unit are shown overlaid, showing both copies have near identical conformations in the crystal. **d,** An overlay of the full TYMV 3′UTR (magenta) with crystal structure 4P5J of the TLS domain alone (cyan). The TLS domain in both structures adopts nearly an identical fold maintaining the tRNA-like classic L-shape configuration. **e,** The TLS+UPD structure (magenta) was aligned with the structure of a serine tRNA extracted from a co-crystal structure of the tRNA bound to its cognate synthetase (yellow; PDB: 1SER). The expanded variable region in serine tRNA forms a stem-loop, but this does not overlap with the position of the TYMV UPD.

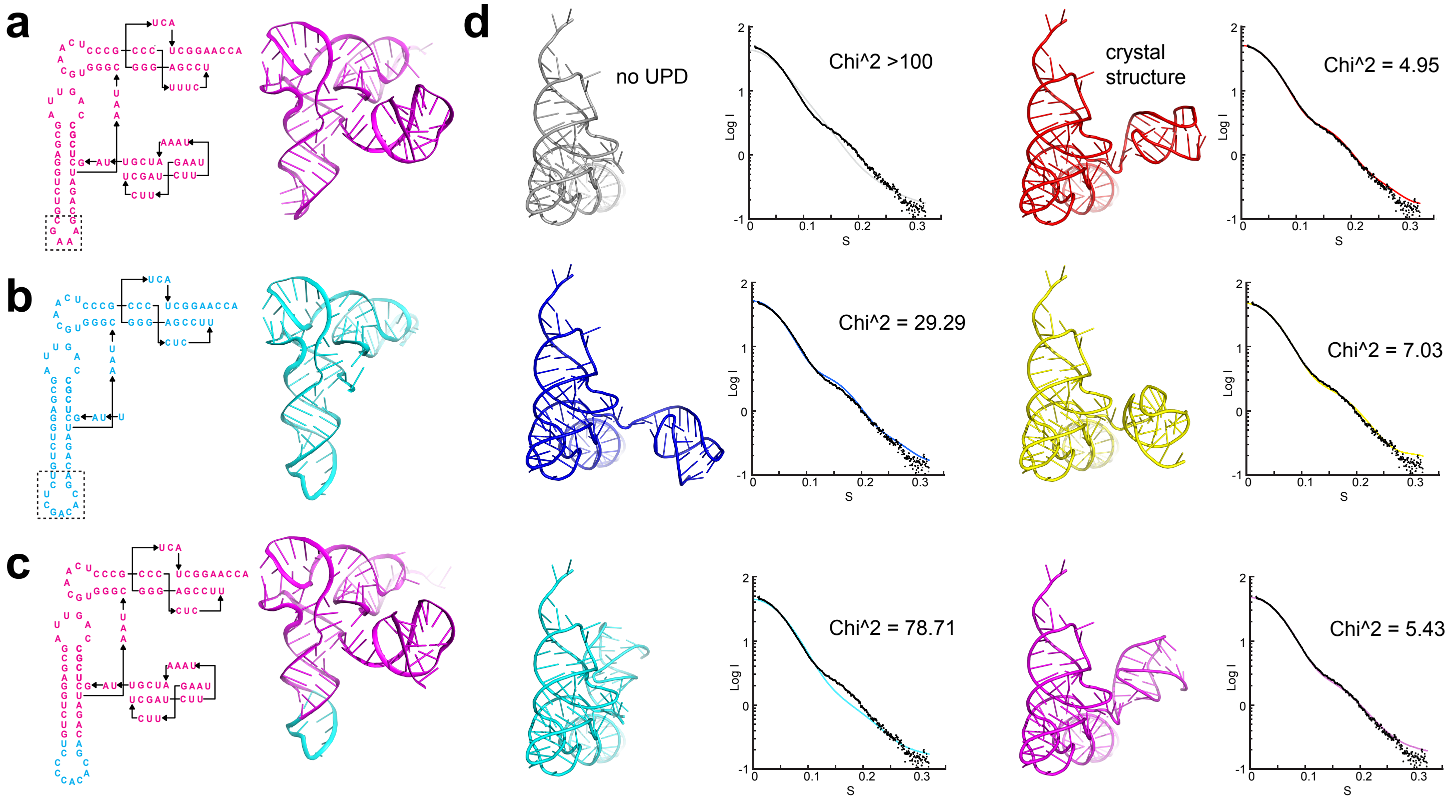

Supplementary Figure 8 Model of the native 3′UTR and SAXS analysis of UPD position relative to the TLS. To generate a model for use in SAXS analysis, we combined the full 3′UTR structure (magenta) and the previously solved crystal structure of the TLS domain (PDB: 4P5J, cyan). **a,** Secondary and tertiary structure of the 3′UTR with the altered anticodon sequence boxed. **b,** Secondary and tertiary structure of the TLS domain solved previously which lacks the UPD, containing the boxed anticodon loop that was used for SAXS experiments. **c,** The composite model (magenta and cyan) was generated by adding the anticodon stem loop from TLS only to the 3′UTR structure. The nucleotides in the PK that were altered for crystallization were also mutated to match the wild-type RNA sequence. **d**, The crystallized conformation of the TYMV 3′UTR matches the solution configuration indicated by SAXS. Previously collected SAXS data using the wild-type TYMV 3′UTR [8] is plotted in grey data points. We constructed models in which the position of the UPD relative to the TLS was altered, and then calculated predicted scattering profiles for each model. These are shown in color and overlaid on the experimental data. The goodness of fit is scored by the Chi-squared value. The grey model (top left) is in the absence of the UPD. The blue model has the UPD extended further away from the TLS. The cyan model has the UPD positioned to pack against the pseudoknot in the TLS. None of these fit the data well. On the right, the yellow and magenta models have the angle between the UPD and TLS altered when compared to the crystallized structure, but with no close packing between the two domains. These better fit the SAXS data, but as indicated by the associated Chi-squared value, the red structure (the crystallized conformation of the TLS and UPD) agrees best with the SAXS data. This indicates that in solution the average ensemble model positions the UPD as observed in the crystal structure, likely without any inter-domain packing.

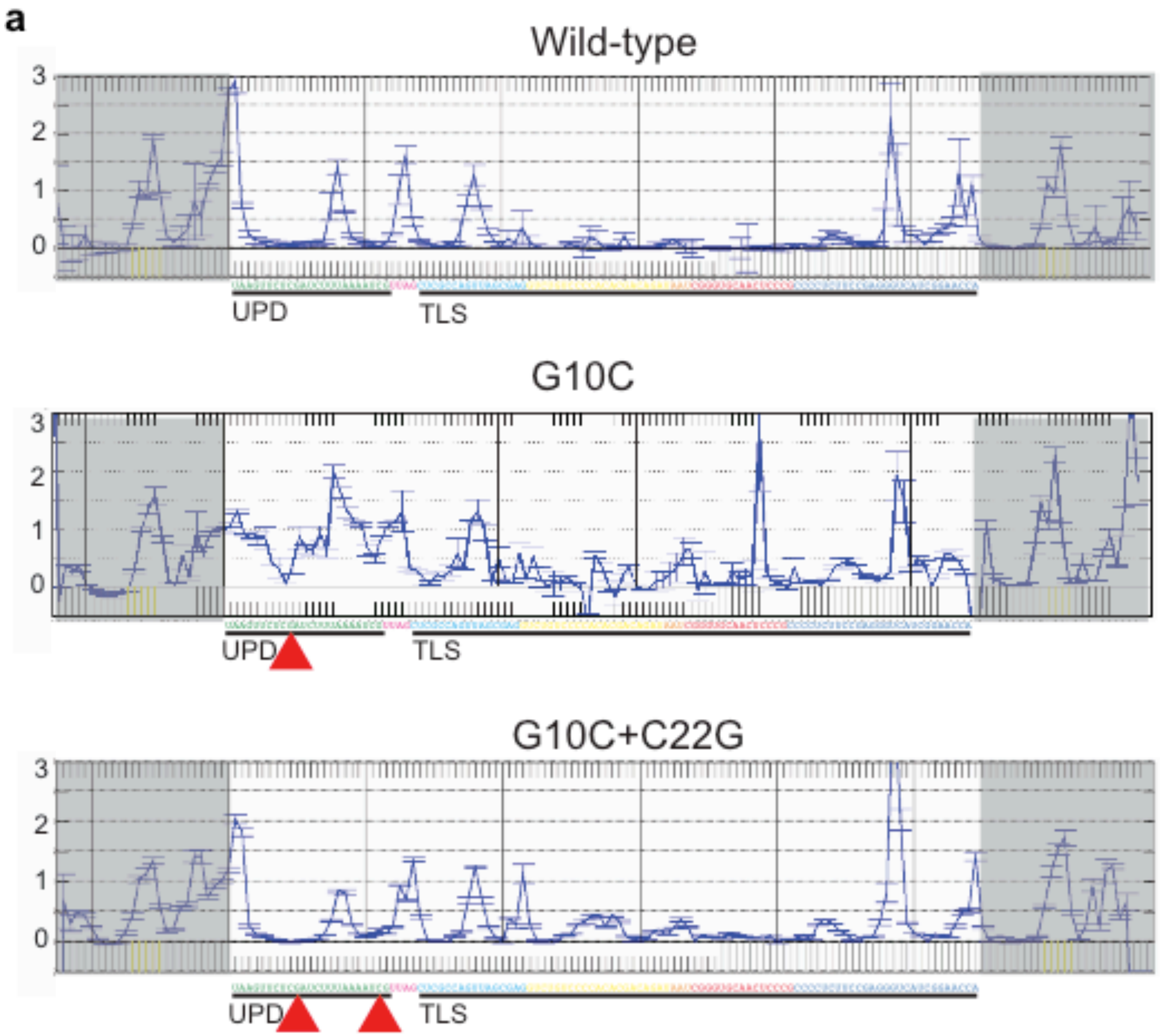

Legend on next page

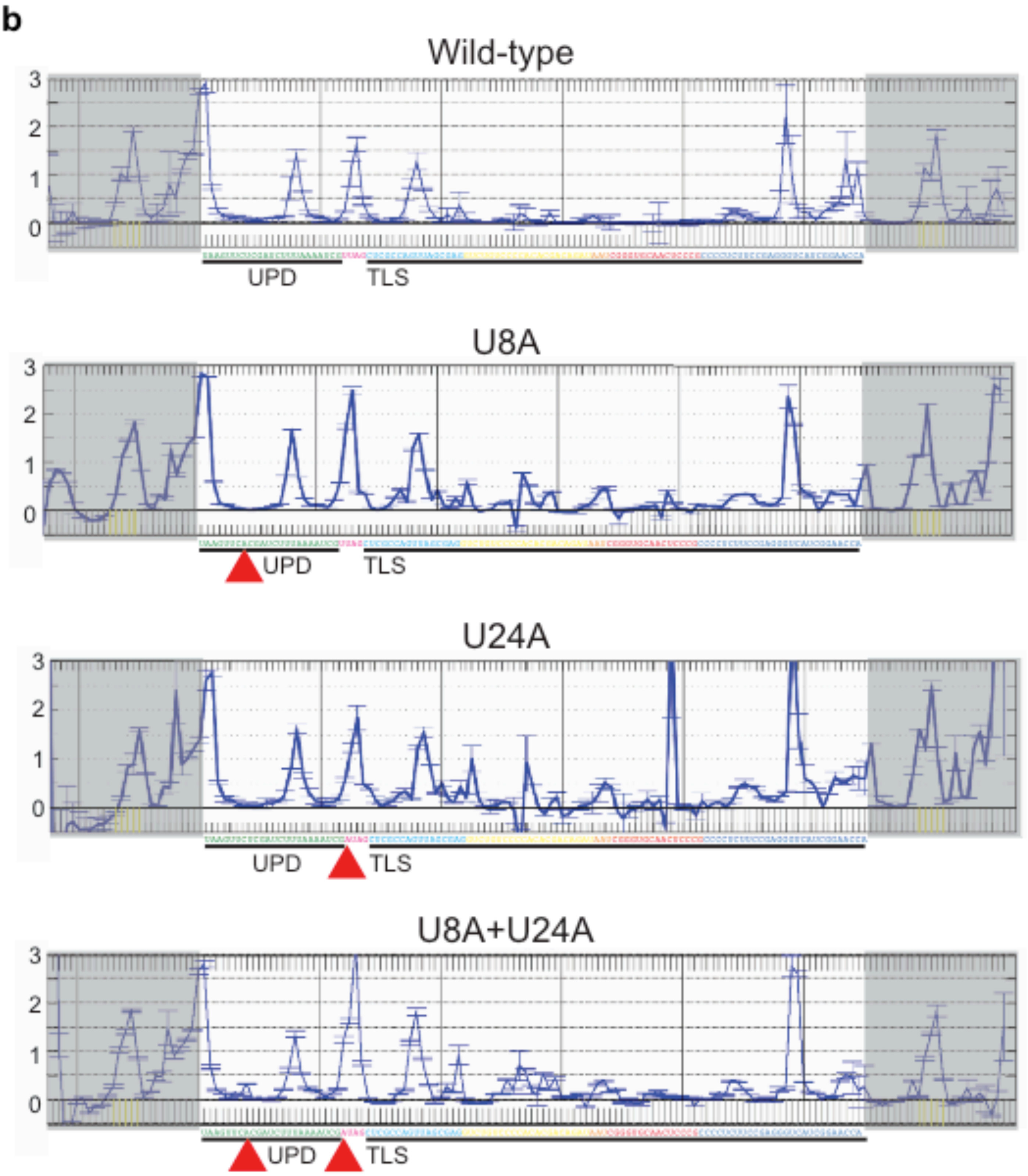

Supplementary Figure 9 Chemical probing of mutant RNAs. **a,** (on previous page) SHAPE (NMIA) probing profiles of the wild-type, G10C and G10C+C22G mutant RNAs are shown. X-axis is position in the sequence colored to match Figure 5. Red triangles mark the location of mutations and the locations of the UPD and TLS are indicated. Grey boxes are regions appended to each RNA to enable quantification of the data. Y-axis is quantitated normalized SHAPE reactivity. **b,** Comparison of the wild-type RNA with the U8A, U24A, and U8A+U24A mutant RNAs. Error bars represent one s.e.m. of n ≥ 3 replicates.

Supplementary Table 1 X-ray data collection and refinement statistics.

**Data collection**	
Space group	I 2 2 2
Cell dimensions	
a, b, c (Å)	103.0, 129.1, 172.0
α, β, γ (°)	90, 90, 90
Resolution (Å)	35.79 - 3.00 (3.18 - 3.0)
R_{sym} or R_{merge}	0.088 (1.69)
R_{pim}	0.035 (0.69)
R_{meas}	0.095 (1.83)
$I/\sigma I$	16.69 (1.37)
CC(1/2)	0.999 (0.82)
Completeness (%)	99.04 (98.28)
Redundancy	7.1 (6.9)
**Refinement**	
Resolution (Å)	35.79 - 3.00 (3.18 - 3.0)
No. reflections	150180 (14427)
R_{work}/ R_{free}	0.241 (0.488) / 0.285 (0.539)
No. atoms	4306
RNA	4306
Ligand/ion	0
Water	0
B-factors	158.85
RNA	158.85
Ligand/ion	0
Water	0
R.m.s deviations	
Bond lengths (Å)	0.009
Bond angles (°)	1.70

Supplementary References

1 Akiyama, B. M. & Stone, M. D. Assembly of complex RNAs by splinted ligation. *Methods Enzymol* **469**, 27-46, doi:10.1016/S0076-6879(09)69002-9 (2009).

2 Spiess, A. N. & Ivell, R. A highly efficient method for long-chain cDNA synthesis using trehalose and betaine. *Anal Biochem* **301**, 168-174, doi:10.1006/abio.2001.5474 (2002).

3 Yoon, S. *et al.* HiTRACE: high-throughput robust analysis for capillary electrophoresis. *Bioinformatics* **27**, 1798-1805, doi:10.1093/bioinformatics/btr277 (2011).

4 Cordero, P., Kladwang, W., VanLang, C. C. & Das, R. The mutate-and-map protocol for inferring base pairs in structured RNA. *Methods in molecular biology* **1086**, 53-77, doi:10.1007/978-1-62703-667-2_4 (2014).

5 Matsuda, D. & Dreher, T. W. The tRNA-like structure of Turnip yellow mosaic virus RNA is a 3'-translational enhancer. *Virology* **321**, 36-46, doi:10.1016/j.virol.2003.10.023 (2004).

6 Sachs, A. B., Davis, R. W. & Kornberg, R. D. A single domain of yeast poly(A)-binding protein is necessary and sufficient for RNA binding and cell viability. *Molecular and cellular biology* **7**, 3268-3276 (1987).

7 Preiss, T., Muckenthaler, M. & Hentze, M. W. Poly(A)-tail-promoted translation in yeast: implications for translational control. *Rna* **4**, 1321-1331 (1998).

8 Hammond, J. A., Rambo, R. P. & Kieft, J. S. Multi-domain packing in the aminoacylatable 3' end of a plant viral RNA. *J Mol Biol* **399**, 450-463, doi:10.1016/j.jmb.2010.04.016 (2010).
